# Sodium arsenite and arsenic trioxide differently affect the oxidative stress of lymphoblastoid cells: An intricate crosstalk between mitochondria, autophagy and cell death

**DOI:** 10.1371/journal.pone.0302701

**Published:** 2024-05-10

**Authors:** Nathan Earl Rainey, Anne-Sophie Armand, Patrice X. Petit

**Affiliations:** 1 CNRS UMR 8003 Paris University, SSPIN, Neuroscience Institute, Team “Mitochondria, Apoptosis and Autophagy Signaling”, Campus Saint-Germain, Paris, France; 2 INSERM U1151, Institut Necker Enfants Malades (INEM), Campus Necker, Université Paris Cité, Paris, France; Texas A& M University, UNITED STATES

## Abstract

Although the toxicity of arsenic depends on its chemical forms, few studies have taken into account the ambiguous phenomenon that sodium arsenite (NaAsO_2_) acts as a potent carcinogen while arsenic trioxide (ATO, As_2_O_3_) serves as an effective therapeutic agent in lymphoma, suggesting that NaAsO_2_ and As_2_O_3_ may act via paradoxical ways to either promote or inhibit cancer pathogenesis. Here, we compared the cellular response of the two arsenical compounds, NaAsO_2_ and As_2_O_3_, on the Burkitt lymphoma cell model, the Epstein Barr Virus (EBV)-positive P3HR1 cells. Using flow cytometry and biochemistry analyses, we showed that a NaAsO_2_ treatment induces P3HR1 cell death, combined with drastic drops in ΔΨm, NAD(P)H and ATP levels. In contrast, As_2_O_3_-treated cells resist to cell death, with a moderate reduction of ΔΨm, NAD(P)H and ATP. While both compounds block cells in G2/M and affect their protein carbonylation and lipid peroxidation, As_2_O_3_ induces a milder increase in superoxide anions and H_2_O_2_ than NaAsO_2_, associated to a milder inhibition of antioxidant defenses. By electron microscopy, RT-qPCR and image cytometry analyses, we showed that As_2_O_3_-treated cells display an overall autophagic response, combined with mitophagy and an unfolded protein response, characteristics that were not observed following a NaAsO_2_ treatment. As previous works showed that As_2_O_3_ reactivates EBV in P3HR1 cells, we treated the EBV^-^ Ramos-1 cells and showed that autophagy was not induced in these EBV^-^ cells upon As_2_O_3_ treatment suggesting that the boost of autophagy observed in As_2_O_3_-treated P3HR1 cells could be due to the presence of EBV in these cells. Overall, our results suggest that As_2_O_3_ is an autophagic inducer which action is enhanced when EBV is present in the cells, in contrast to NaAsO_2_, which induces cell death. That’s why As_2_O_3_ is combined with other chemicals, as all-trans retinoic acid, to better target cancer cells in therapeutic treatments.

## Introduction

Arsenic (As) is a natural element that exists in groundwater in highly toxic inorganic forms, mainly pentavalent arsenic (arsenate, As^5+^) and trivalent arsenic (arsenite, As^3+^), the latter being more toxic and mobile than the pentavalent form. Among the trivalent arsenic, two of them are of particular interest in the cancer field: sodium arsenite (NaAsO_2_) and arsenic trioxide (ATO, As_2_O_3_). While both are toxic for the cells, studies on NaAsO_2_ have mainly focused on its carcinogenic effect [1, 2], and ATO has been highlighted for its anticancer properties [3].

At a biochemical level, NaAsO_2_ can replace phosphate in several cellular reactions. The arsenic oxyanion exhibits similarities with the phosphate ion, and part of its toxicity could be understood as a competitive inhibition towards phosphate-utilizing enzymes, found in intermediate metabolism and oxidative phosphorylation [4, 5]. On the other hand, ATO reacts with thiols (-SH) in proteins and inhibits their activity. Other mechanisms include epigenetic alteration, oxidative stress, inflammation, and autophagic defects [6–8]. Sodium arsenite forms relatively weak bonds with monothiols, and high intracellular concentrations of arsenite can deplete cells of glutathione (GSH). It forms strong bonds with dithiols in small molecules such as the lipoic acid cofactor and with vicinal thiols in proteins, leading to inactivation of various enzymes and receptors. Unlike arsenate, which was long known to be taken up by phosphate transporters, the way for more toxic arsenite (like ATO or trivalent methylated arsenicals) to enter the cell was unknown until the ground-breaking study by Sanders *et al*. [9] showing that these molecules enter via the glycerol facilitator (as they are recognized as polyols).

Once in the cell, arsenic induces formation of O_2_^.-^, H_2_O_2_, ^-^OH, and ROO^-^. Formation of O_2_^.-^ and H_2_O_2_ in response to arsenic exposure in different cell lines is summarized by Shi *et al*. [10]. Arsenic induces generation of ROS by several mechanisms: (*i*) Mitochondria: Mitochondrial complexes I and III in the electron transport chain are responsible for the production of O_2_^.-^. Arsenic shows mitochondrial toxicity by inhibiting succinic dehydrogenase activity and uncoupling oxidative phosphorylation with production of O_2_^.-^, which gives rise to other forms of ROS [11]; (*ii)* Nicotinamide adenine dinucleotide phosphate (NAD(P)H) oxidase (Nox): Nox is a membrane-associated enzyme involved in ROS generation in response to arsenic [12]; (*iii*) Generation of ROS during formation of intermediate arsine species [13, 14]; (*iv*) Redox-active iron [15] released from ferritin caused by methylated arsenic species [16]; (*v)* Endoplasmic reticulum (ER): ER is suggested to be a source of ROS caused by dimethylarsinous acid (DMA III), which is a metabolite of NaAsO_2_ [17, 18]; (*vi*) Interference with cellular antioxidants: superoxide dismutase (SOD), catalase (CAT) [19], glutathione (GSH) [20], and GSH-related enzymes [21], which indirectly result in increased ROS levels and therefore oxidative stress induction, leading to many cellular defects potentially inducing cancers.

Hematopoietic malignancies, such as Burkitt lymphoma, post-transplantation lymphoproliferative disorder, diffuse large B-cell lymphoma and acute promyelocytic leukemia (APL) have long been associated to the Epstein-Barr virus (EBV). The current approach to EBV-associated lymphoma usually involves chemotherapy to eradicate cancer cells. However, healthy cells may be injured and organ dysfunction may occur with the regimens currently employed. Treatment of APL has become a paradigmatic example of modern precision medicine [22]. The introduction of the chemical combination of all-trans retinoic acid (tretinoin or ATRA) together with ATO has converted APL from being an extremely lethal disease to a highly curable one [23], with a relative high efficacy [24–27]. This success represents an emblematic shift towards lower exposure to chemotherapeutic agents, and hence a reduction usually in both acute and long-term side effects. The new oral arsenic formulations may allow better compliance in younger patients and will further minimize the potential side effects of intravenous agents. Importantly, the safer toxicity profile of oral vs. intravenous arsenic formulations may also be a key to reduce the hospital stay.

Despite their high efficacy, the early use of ATRA and ATO [28] has been tested without a complete understanding of their modes of action. In most cases, APL is characterized by the fusion of the promyelocytic leukemia (*PML*) gene on chromosome 15 with the retinoic acid receptor alpha (*RARα*) gene on chromosome 17 [29]. The resulting fusion protein, PML-RARα, induces both a block of the cell differentiation at the promyelocytic stage [30] and a cell survival and proliferation [31]. On a mechanistic point of view, ATRA has been shown to interact with PML-RARα and reverse the transcriptional repression of the fusion protein, allowing cell differentiation [32]. More recently, ATRA has been described as a potent disruptor of lipid homeostasis, with an early decrease in mitochondrial cardiolipin, associated with inhibition of mitochondrial activity [33]. The biological relevance of non-genomic effects of retinoids (e.g. phosphorylation, membrane effects) is still under investigation [34].

As ATRA, ATO interacts with the PML-RARα fusion protein, but this binding induces the degradation of PML-RARα via SUMOylation and ubiquitylation [35, 36], resulting in partial differentiation and induction of cell death of leukemic promyelocytes. However, the literature concerning inorganic arsenicals is still controversial in terms of the signal transduction pathways leading to cancer cell death.

Here, we investigated the cell death signaling pathways induced by ATO treatment and compared them to those activated by the highly carcinogenic arsenic chemical form, sodium arsenite (NaAsO_2_), in a Burkitt lymphoma cell model, the EBV-positive (EBV^+^) P3HR1^+^ cells. By using multiparametric flow cytometry analysis and image cytometry, we analyzed the cellular response induced by the two arsenic compounds. Events were investigated with careful attention paid to ROS production levels, balancing pathways between autophagy and cell death. Also, we specially focused on cell fate according to EBV status, by comparing the cellular response of a non-cancer cell line, the B lymphocyte Ramos-1 cells, which are EBV negative to the two arsenic compounds.

## Materials and methods

### Cell lines

We used Ramos-1 cells, B lymphocytes, EBV^-^ cells (ATCC^®^ CRL-1596^™^) and P3HR1, EBV^+^ cells (ATCC^®^ HTB-62) from Burkitt lymphoma, where Herpes-type virus particles (EBV) were observed in as many as 78% of the cells examined with the electron microscope and a good correlation was noted between the presence of viral particles and immunofluorescence.

### Reagents, cell lines, and culture conditions

Sodium arsenite (NaAsO_2_) and arsenic trioxide (As_2_O_3_) were from Sigma-Aldrich (St. Louis, MO). Fresh stock solutions of 2 mM NaAsO_2_ and As_2_O_3_ were prepared before every experiment and filter sterilized using a 0.2 μm syringe filter. RPMI-1640, penicillin, and streptomycin were from GIBCO. 5-chloromethylfluorescein diacetate (5-CMF), DAPI, DCFH-DA, DiOC_6_(3), Fluo4-AM, MitoSOX red, YO-PRO-1, TO-PRO-3 and propidium iodide (PI) were from Molecular Probes (Invitrogen, Thermo Fisher Scientific).

The P3HR1 or Ramos cell lines were grown in suspension in RPMI 1640 with 10% heat-inactivated fetal calf serum, at 37°C in a 5% CO_2_ atmosphere. When working on cellular bioenergetics, 5 mM Na-pyruvate was added to the culture medium. Cells were sub-cultured at 1:2 ratios every 2 to 3 days with a viability greater than 95% (assessed by trypan blue and also by YOPRO-1/PI staining) maintaining cell concentrations between 0.5 and 1.5 x10^6^/mL.

### Cell death assessment

P3HR1 or Ramos cells were decanted in 24-well culture plates at 0.5 10^6^/mL. RPMI was added as untreated control. The plates were incubated for various times from 0 to 72 h at 37°C with 5% CO_2_. To identify dead and live cells, samples were labeled with YO-PRO-1 and PI. Briefly, samples were incubated in 1 μM YO-PRO-1 in RPMI for 15 min, washed three times in phosphate buffer saline by centrifugation, transferred to flow cytometry tubes, stained with 1 μM PI, and immediately analyzed with a Becton-Dickinson FACSCalibur 4C flow cytometer with excitation at 488 nm. A total of 10,000 cells were analyzed in each sample. YO-PRO-1 and PI fluorescence values were collected through 535 ± 15 nm and 680 ± 15 bandpath filters, respectively. Quadrant analysis was done with our own setting adapted to each situation.

### Cell size or volume change

The mean volume and diameter of cells treated with 5 M of either arsenical compound for 24, 48 and 72 h were determined on a Cell Counter and Analyzer CASY TT (Schärfe System Casy TT, Germany). Cell scattering characteristics obtained by flow cytometry analysis were collected as a measurement of the side scatter (SSC) and low-angle light scatter (FSC) of intact cells taken from the 488 nm excitation laser during the first hour of the treatment, before apoptosis and/or secondary necrosis of the cells (the experiments were done with a Becton-Dickinson FACSCalibur 4C).

### Determination of mitochondrial membrane potential (ΔΨm), reactive oxygen species and phosphatidylserine residues exposed at the plasma membrane

A density of 2x10^6^ P3HR1 or Ramos-1 cells on 6-well plates were maintained with no chemical or 5 μM NaAsO_2_ or 5 μM As_2_O_3_ for a given time ranging from 0 to 72 h depending on the experiments. After treatment, cells were trypsinized and then harvested, washed, and resuspended together with their supernatant in PBS (in order to collect the dead cells). 3,3’-Dihexyloxacarbocyanine-iodide [DiOC_6_(3)] was added at 40 nM final concentration for ΔΨm determination, 2’,7’-dichlorodihydrofluorescein diacetate (DCFH-DA) at 5 μM for hydrogen peroxide measurement, and MitoSOX at 1 μM for superoxide anions. Double staining was mostly done in order to assay simultaneously cell viability, with PI (1 mg.mL^-1^ stock solution) for DiOC_6_(3), DCFH-DA and with TO-PRO-3 iodide (1 mg.mL^-1^ stock solution) for MitoSOX. For the ΔΨm determination, fluorescence of a positive control was elicited by exposing cells to 10 μM carbonyl cyanide m-chlorophenyl hydrazone (CCCP; Sigma-Aldrich) 10 min prior to adding the DiOC_6_(3) probe, and basal fluorescence of non-treated samples was used as reference to compare changes induced by single or combined drug treatment [37].

A supplemental double staining was used for the distinction between viable, apoptotic, and necrotic cells with YO-PRO-1 iodide/PI (Y3603, for YOPRO-1, Molecular Probes) in parallel with annexin-V/PI staining done with annexin-V-FITC when needed (Immunotech, Beckman-Coulter) in the presence of calcium in order to detect the aberrant exposure of phophatidylserine residues at the outer surface of the plasma membrane. All samples were analyzed using flow cytometry as previously described [38] on a FACSCalibur 4C.

### NAD(P)H determination by flow cytometry

NAD(P)H fluorescence was elicited with a multiline ultraviolet light set at 400 mW on a FACS Vantage. Changes in the autofluorescence of normal and apoptotic cells were recorded as previously described [38].

### Antioxidant determination

Glutathione (GSH) levels were detected with 5-chloromethylfluorescein diacetate (5-CMF), a probe that forms fluorescent adducts with intracellular non-protein thiols [39, 40]. More than 95% of these adducts correspond to GSH-5-CMF adducts [41]. After treating cells with single drugs for the appropriate duration (0-72h), they were incubated in serum-free medium for 15 min in 0.5 μM 5-CMF, then washed by centrifugation, and stained with 1 μM PI to further exclude any dead cells from the analysis.

Superoxide dismutase (SOD) measurement: total SOD activity was determined using a commercial kit for which the activity of SOD was defined by its ability to inhibit the oxidation of hydroxamine hydrochloride to nitrite with superoxide. Briefly, 10^6^ cells were exposed to the desired concentrations of NaAsO_2_ or As_2_O_3_ at various times in 6-well plates. Cells were collected by trypsinization, followed by lysis and centrifugation at 900 ~g for 5 min, and the supernatant was analyzed according to the manufacturer’s instructions using the Superoxide Dismutase (SOD) Activity Assay Kit (Clinisciences, France). The absorbance was measured at 500 nm using a spectrometer. Fifty percent inhibition was defined as one unit enzyme activity and normalized to the protein concentration.

### Determination of lipid peroxidation, protein carbonylation and lactate production

We used a lipid peroxidation assay kit (Abcam) to detect malonaldehyde (MDA) present in samples. The free MDA generated during lipid peroxidation refers to the oxidative degradation of lipids reacting with thiobarbituric acid (TBA) to generate MDA-TBA adducts. The absorbance of TBA-MDA adduct was measured at 532 nm and this kit detects levels as low as 1 nmol/well. For the calculation, we determined the MDA concentration in standards and samples from their absorbance, as described in the protocol of the lipid peroxidation assay kit from Abcam (ab118970). Protein carbonylation in P3HR1+ cell lysates was assayed using Cayman’s Protein Carbonyl Fluorometric Assay Kit. Cytotoxicity was assessed by measuring the release of lactate dehydrogenase (LDH) into the medium using the CytoTox96 LDH assay kit (Promega, France). Culture supernatant (50 mL) was incubated with an equal volume of LDH substrate solution in dark conditions for 30 minutes. The reaction was stopped with 50 mL of 1 M acetic acid, and the absorbance was determined at 492 nm. For lipid peroxidation, protein carbonylation and lactate production, at least 10 different separate experiments were carried out, unless otherwise indicated.

### Determination of caspase activity

Controls and treated cells were plated in 6-well plates at a density of 2.10^6^ cells/well and incubated for various times (0 to 48 h) with the appropriate arsenical compound and then harvested. The samples were then washed, resuspended in 50 μL of 10 μM substrate solutions (PhiPhiLuxG1D2 for caspase-3, CaspasLux8 for caspase-8 and CaspasLux9 for caspase-9), and incubated at 37°C for 1 h according to the assay manufacturer’s (Oncoimmun, USA) instructions. The cells were then washed again with PBS and analyzed by flow cytometry. The results are presented as the percentage of cells testing positive at each staining, but the F mean of fluorescence of the positive population is also given.

The basic control level for each caspase is around 100 ± 9 (F mean, a.u.) for laser excitation at 488 nm with a PMT value of 350. The samples were analyzed on a flow cytometer using a 530 nm bandpass (for the caspase activity) and 670 nm long-pass emission filters for PI (exclusion of the dead cells fully permeable to PI). * Percentage of cells with a given activity.

### Annexin V-FITC/PI staining assay

To evaluate the surface-exposed phosphatidylserine on cells, which is an early indication of apoptosis, the Annexin V- FITC Apoptosis Detection Kit (KeyGen Biotechnology, Nanjing, China, Cat. No. KGA108) was utilized. In this kit, annexin V and PI were used to distinguish the apoptotic and necrotic cells. According to the manufacture’s protocol, the exponentially proliferating cells were exposed to the designated concentrations of NaAsO_2_ and As_2_O_3_ in 6-well plates at a density of 10^6^ cells/well for 24 h. Cells were washed in cold PBS. Thereafter, cells were centrifuged at 1200 rpm for 5 min at 4^◦^C, resuspended in 400 μL binding buffer containing 2 μL of FITC-conjugated annexin V and 1 μg/mL PI. After incubation in the dark at 37^◦^C for 30 min, the cells were measured by a flow cytometry (FACSCalibur 4C).

### Analysis of CHOP and GRP78 activities by flow cytometry

P3HR1^+^ cells were for treated 48 h with 5 μM As_2_O_3_ or 5 μM NaAsO_2_ and tested for GRP78 and CHOP abundance. Cells were grown in 6-well plates, trypsinized and then fixed with 4% paraformaldehyde at 4°C for 40 min and rinsed several times with PBS. Nonspecific binding sites were blocked for 2 h at room temperature with 5% normal SVF (Gibco, ThermoFisher Scientific, Waltham, MA, USA) in 0.1% Triton X-100-PBS. Caco-2 cells were incubated overnight at 4°C with primary antibodies (1:100 dilutions with blocking buffer) for GRP78 (Cell Signaling, Danvers, MA, USA) or CHOP (Santa Cruz, CA, USA). Cells were then incubated with the appropriate fluorescein isothiocyanate or tetramethylrhodamine isothiocyanate-conjugated secondary antibodies (BD Biosciences, Grenoble, France) for 2h at 4°C. Cells were analyzed by flow cytometry using the green (FL1 = 530 ± 30 nM) or red (FL-2 = 585 ± 42 nm) channels. Each experiment was repeated at least four times in duplicate.

### Cell cycle analysis by flow cytometry

P3HR1^+^ or Ramos-1 cells were taken from the culture wells at defined times of incubation with or without the proper chemicals and their position in the cell cycle was evaluated by measuring 5-bromo-2-deoxyuridine (BrdU) incorporation using the APC BrdU Flow Kit (catalogue number 552598, BD Pharmingen). Cells were incubated with BrdU (10 mM) for 1 h at 37° C, washed, trypsinized, and fixed with cytofix/cytoperm buffer. BrdU staining was done following the kit procedure. DNA was stained with 7-aminoactinomycin D (7-AAD) and cells were analyzed using a FACSCalibur4C (Becton Dickinson) with the FL-1 channel (530 ± 30 nm band pass) for BrdU and the FL-3 channel (670 nm long pass) for 7-ADD. The sub-G_0_G_1_ peak represented the dead cells within the samples.

### Acridine orange staining of the acidic compartment

Acridine orange (AO; A-3568, Molecular Probes, Invitrogen) was used as a reporter of autophagic vacuole formation. It crosses lysosomal membranes (and other acidic compartments) and becomes protonated [54]. The protonated dye stacks and stacked AO emits in the red range. If AO is not in an acidic compartment, its emission is in the green range. It has to be taken into account that when taken as a DNA intercalator, for example, its fluorescence intensity decreases when DNA condenses (that is the case when cells pass from an autophagic status and undergo autophagic cell death). As a marker of autophagy, the volume of the cellular acidic compartment was visualized by AO staining [42–44]. Cells were seeded in six-well tissue culture dishes and treated as described above for the cell viability study. Cells were incubated with medium containing 1 μg/mL AO for 15 minutes. Cells were washed twice with PBS to remove excess AO, fresh medium was added, and fluorescence micrographs were taken using an Olympus inverted fluorescence microscope. All images presented are at the same magnification. The number of cells with increased acidic vesicular organelles was determined by flow cytometry [43, 44]. Cells were trypsinized, harvested and analyzed by BD FACSCalibur 4C (using Cellquest software). A minimum of 10,000 cells within the gated region were analyzed. We bore in mind that acidotropic dyes like AO only stain late autophagic vacuoles.

### Image stream examination of lysosomal acidification combined with direct immunofluorescence LC3B labeling and also mitochondrial membrane potential staining by TMRE combined with direct immunofluorescence LC3B labeling

Imaging flow cytometry combines the strengths of microscopy and flow cytometry in a single system for quantitative image-based cellular assays in large and heterogeneous cell populations. The Amnis Image Stream 100 instrument (Amnis, Merck Millipore, Burlington, MA, USA) acquires up to six images of each cell in different imaging modes. The system is equipped with 3 lasers (405 nm, 488 nm and 640 nm) and cells can be magnified by a 20, 40 or 60 X objective, allowing a wide range of applications. The given images of each cell comprise: a side-scatter (SSC) image, a transmitted light (brightfield) image and fluorescence images corresponding to the FL1, FL2, FL3 and FL4 spectral bands of conventional flow cytometers [45].

P3HR1^+^ cells with or without treatment were pelleted and resuspended in 100 μL of Solution A fixative for 15 min at room temperature (RT; cat. no. GAS‐002A‐1, Caltag, UK) and then washed in PBS buffer. Then the cells were stained with LysoTracker Deep Red to estimate the lysosomal acidification, following the manufacturer’s instructions (C1046, Molecular probes, Invitrogen), for at least 1 h at 37°C with 1 μM LysoTracker Deep Red. Cell pellets were then permeabilized with 0.25% Triton X‐100 (cat. no. X100–500ML, Sigma Chemicals) for 15 min at RT. Anti‐LC3B polyclonal antibody (0.25 μg; cat. no. L10382, Invitrogen) or rabbit immunoglobulin (0.25 μg; cat No I5006, Sigma Chemicals) was used as an isotype control and incubated for 0.5 h at room temperature. Cells were again washed in PBS buffer and then labeled with 0.125 μg of secondary fluorescent conjugate Alexa Flour 488 goat anti‐rabbit IgG (cat. no. A11034, Invitrogen) for 30 min at RT. Cells were then washed in PBS buffer and resuspended in 400 μL of PBS in the presence of DNA viability dye, DAPI (200 ng/mL). The LC3B‐Alexa Fluor647 signal was analyzed by determining the MFI of the whole histogram signal for previously live cells gated from an FSC versus SSC dot‐plot. LC3B MFI upregulation from autophagy‐induced samples and untreated cells was compared to show the degree of induced autophagy. Average fold increase for autophagic samples was calculated from average control MFI LC3B levels and the individual data points shown (*n* = 5). LC3B‐labeled samples were also compared to corresponding isotype control samples in an overlaid histogram. About 500 to 1000 events were collected [46]. In case of mitochondrial membrane potential concern, the fixable tetramethyl rhodamine ester probe (TMRE) has been used at 40 nM together with LC3B ‐Alexa Fluor647. For the colocalization analysis, the Similarity Score included in IDEAS 6.0 software™ (Amnis). This score, a log-transformed Pearson’s correlation coefficient between the pixels of two image pairs, provides a measure of the degree of co-localization by measuring the pixel intensity correlation between the LysoTracker images and the LC3B-647 images and/or between the TMRE images and The LC3B-647 images. In case of TMRE a false green color has been used instead of orange/red fluorescence of the dye for a more comfortable reading of the images.

### Real-time PCR

Real-time PCR reactions were performed in duplicate using Takyon™ No Rox Probe MasterMix dTTP blue (Eurogentec) on a 7900HT Fast Real-Time PCR System (Applied Biosystems). Transcripts were quantified using the following program: 3 min at 95°C followed by 35 cycles of 15 s at 95°C, 25 s at 60°C and 25 s at 72°C. Values for each transcript were normalized to expression levels of RPL13A (60S ribosomal protein L13a) using the 2^-^ΔΔ^Ct^ method. Primers used for quantification of transcripts by real-time quantitative PCR are the following:

RPL13A-Forward: 50-CCTGGAGGAGAAGAGGAAAGAGA-30,
RPL13A-Reverse: 50-TTGAGACCTCTGTGTATTTGTCA-30,
LC3B-Forward: 50-GGCCTTCTTCCTGTTGGTGAA-30,
LC3B Reverse: 50-TCTCCTGGGAGGCATAGACCA-30.

### Transmission electron microscopy

Transmission electron microscopy services, including sample fixation, embedding, ultra-microtomy and staining were provided by the VCU Department of Anatomy and Neurobiology Microscopy Facility. Sections were imaged via a Jeol JEM-1230 transmission electron microscope (EM) equipped with a Gatan UltraScan 4000SP 4KÅ~ 4K CCD camera. The magnification of each image is indicated by the scale bar at the bottom of the micrograph.

### Statistics

Statistical analysis has been carried out with the Kruskal Wallis test (*p < 0.05; **p < 0.01; ***p < 0.001). Data are expressed as mean ± SD with the number of experiments cited as n.

## Results

### • As_2_O_3_ and NaAsO_2_ differentially affect P3HR1^+^ cell viability and cell structure characteristics

**Fig 1:**
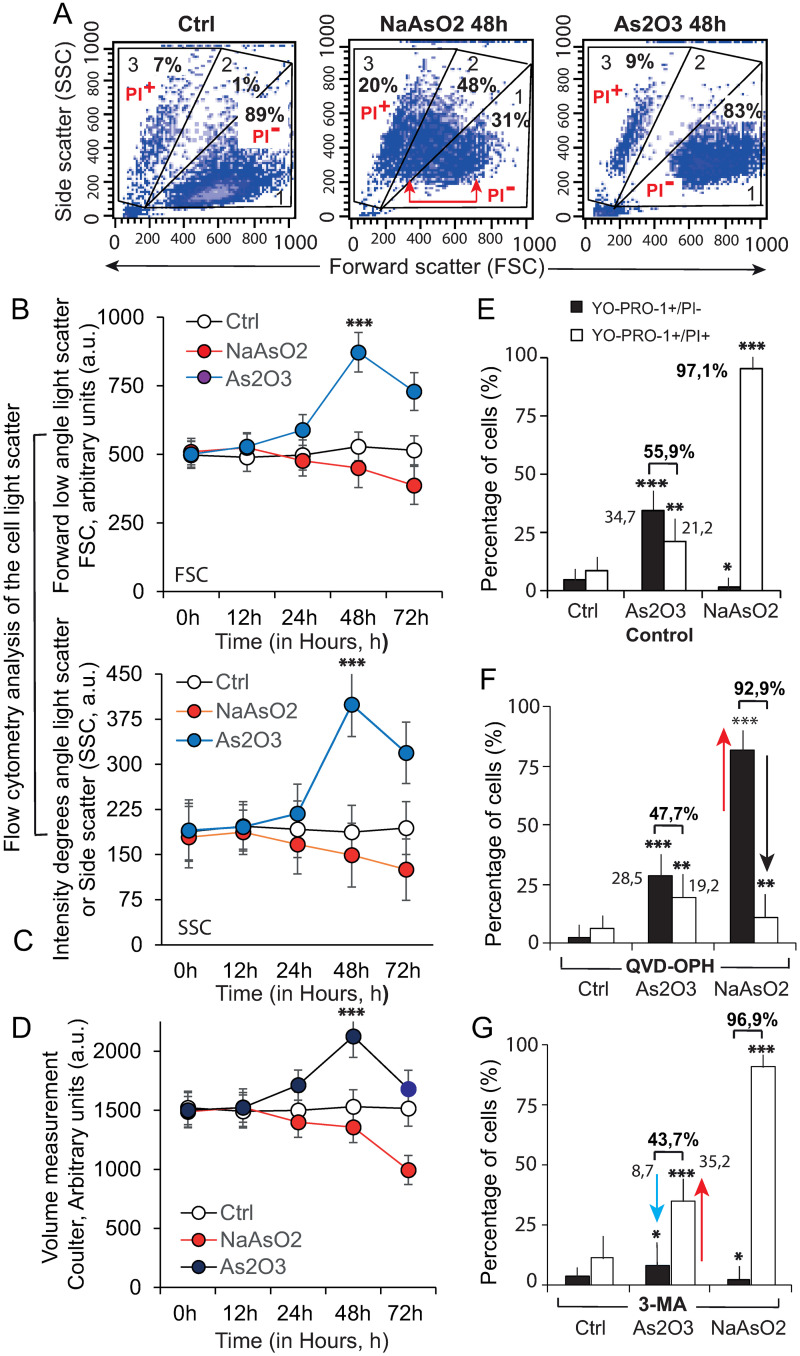
As_2_O_3_- and NaAsO_2_-treated P3HR1^+^ (EBV^+^) cells undergo distinct cell death processes. A–Proof of concept: Cellular swelling associated with the treatment of P3HR1^+^ cells by 5 μM As_2_O_3_ or NaAsO_2_ as evaluated by flow cytometric light scattering analysis using forward light scatter (FSC) and 90° angle light scatter (side scatter, SSC) of the treated P3HR1^+^ cells in complete medium alone (Control) or treated for 48 h with 5 μM As_2_O_3_ or NaAsO_2_. Three different populations are defined regarding FSC and SSC: 1—Viable cells that are PI-negative, YO-PRO-1-negative—2—Intermediate cells that are PI-negative but permeable to YO-PRO-1 and—3—Dead cells are PI-positive and YO-PRO-1-positive. B and C—P3HR1^+^ cells were cultivated for 12, 24, 48 or 72 h in complete medium alone (Control) or containing 5 μM As_2_O_3_ or NaAsO_2_ and further analyzed by flow cytometry for their light scattering properties at 488 nm excitation. In B—Measurement of their forward low angle light scatter (FSC) and in C—Measurement of their scatter at ninety degrees (“side-scatter”, SSC). The experiments have been repeated with n = 10. D—P3HR1^+^ cells were cultivated for 12, 24, 48 or 72 h in complete medium alone (Control) or containing 5 μM As_2_O_3_ or NaAsO_2_. The quantification of mean cell volumes was performed on a Coulter Counter and Analyzer, as described in Materials and Methods. All the statistics have been taken from at least n = 8 separated experiments. For B, C and D. Asterisks indicate statistically significant variation compared to the corresponding population in control cells, calculated using Student’s t-test (*P < 0.01, **P < 0.001). E—P3HR1^+^ cells were treated with 5 μM of As_2_O_3_ or NaAsO_2_ for 72 h and stained with YOPRO-1/PI to determine their viability, that is the sum of YOPRO-1^+^/PI^-^, i.e., apoptotic cells + YOPRO-1^+^/PI^+^, necrotic cells (or “secondary necrosis”). All the statistics have been taken from at least n = 8 separated experiments. F and G—P3HR1^+^ cells were treated with 5 μM of As_2_O_3_ or NaAsO_2_ for 72h in presence of either the pan-caspase inhibitor QVD-OPH (Quinoline-Val-Asp-Difluorophenoxymethyl Ketone, 10 μM) that is blocking caspase-3, -7, -8, -9, -10 and -12 or the autophagy inhibitor 3-methyladenine (3-MA, 5 mM). The arrows are in red for necrotic changes (YOPRO-1^+^/PI^+^) and in black when it is concerning changes in apoptosis (YOPRO-1^+^/PI^-^). Asterisks indicate statistically significant variation compared to the corresponding population in control cells, calculated using Student’s t-test (*P < 0.01, **P < 0.001). All the statistics have been taken from at least n = 8 separated experiments.

We first estimated some basic events affecting the P3HR1^+^ cells when treated with 5 μM As_2_O_3_ or NaAsO_2_: cellular volume changes were assayed by Coulter volume measurement and viability by a flow cytometry analysis combining YO-PRO-1 and PI, and also light scattering measurements (Fig 1).

The light scattering pattern of treated cells differs dramatically depending on the treatment. Arsenic trioxide at 48 h clearly induces cellular swelling (83% of the cells) without significant induction of dead cells (only 9%) (Fig 1A, right panel), whereas the cells treated with sodium arsenite undergo a more complex process with 20% dead cells, 48% of the cell population with increased 90° light scatter (SSC) but slightly decreased forward scatter (FSC), and 31% of the cellular population still unaffected (Fig 1A, central panel) in terms of light scattering. The time course of the light scattering variation along the time of drug exposure confirmed maximal swelling of the cell population with As_2_O_3_ at 48 h when slight shrinkage was observed with NaAsO_2_ (Fig 1B and 1C). This cellular behavior is also confirmed when testing for volumetric changes (Fig 1D).

To get a finer measurement of cell viability, we stained the cells with both YOPRO-1 and PI, two different DNA stain with different sizes, and therefore a differential cell penetration, detecting a small insult to the plasma membrane (YO-PRO-1 staining) or greater permeability (PI staining). The results of the treatment of P3HR1^+^ cells with 5 μM As_2_O_3_ or NaAsO_2_ for 48 h are clearly different. An apoptotic-like population with early membrane permeabilization (YOPRO-1^+^/PI^-^, 34,7%) was seen with the As_2_O_3_ treatment, together with a small proportion of dead cells (YOPRO-1^+^/PI^+^, 21,2%) (high membrane permeability) (Fig 1E), whereas NaAsO_2_ treatment killed 97.1% of the treated cells (YOPRO-1^+^/PI^+^) (Fig 1E). Since the involvement of caspases has been described in the recent literature for As_2_O_3_ [15] and NaAsO_2_ [47], we were curious about a possible effect of a pan-caspase inhibitor (QVD-OPH, quinoline-Val-Asp-difluorophenoxymethylketone) on the cell death pathway we have detected (Fig 1F). It is clear that the QVD-OPH caspase inhibitor only slightly reduced the amount of apoptotic or dead cells and did not change the ratio of apoptotic to dead cells (around 47.7% of cells instead of 55.9%) in As_2_O_3_-treated cells, whereas it efficiently abrogated cell death in the NaAsO_2_-treated cells (Fig 1E and 1F), suggesting that the As_2_O_3_ induced cell death follows a caspase-independent mechanism.

To estimate the possible modulation of cellular viability by autophagy, we treated the P3HR1^+^ cells with the autophagy inhibitor 3-MA, which inhibits autophagosome formation (Fig 1G). Autophagy inhibition clearly induced cells defined as YO-PRO-1^+^/PI^-^ (less than 10%) to shift towards a YO-PRO-1^+^/PI^+^ profile (up to 35%) when cells were treated with As_2_O_3_, showing that autophagy is probably involved in As_2_O_3_-induced resistance to cell death. However, 3-MA had no influence on the cell death (96.9% of cells) induced by NaAsO_2_ (Fig 1G).

### • As_2_O_3_ and NaAsO_2_ affect mitochondrial bioenergetics in different ways

**Fig 2:**
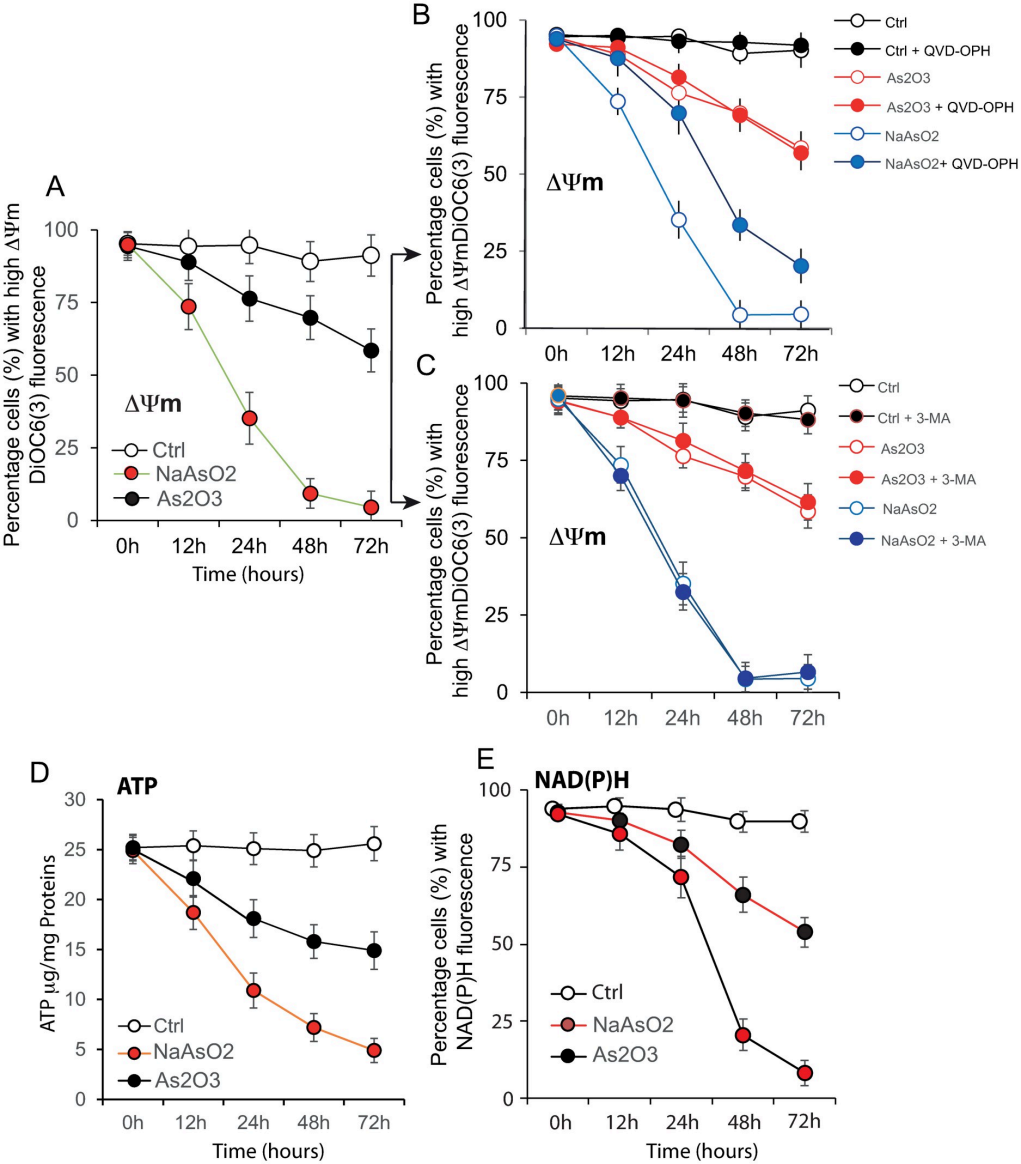
Cellular activities and cell cycle analysis affected by the As_2_O_3_ or NaAsO_2_ treatment. A–Malondialdehyde production at 0 h and 48 h for P3HR1^+^ and Ramos-1 cells treated with either 5 μM As_2_O_3_ or 5 μM NaAsO_2_ (n = 7). (B) Protein carbonylation in P3HR1^+^ and Ramos-1 cells treated with 5 μM As_2_O_3_ or 5 μM NaAsO_2_ (n = 8). (C) Lactate production induced by 48-h treatment with 5 μM As_2_O_3_ or 5 μM NaAsO_2_. Data are expressed as nmol/mg protein (n = 8). (D, E)—P3HR1^+^ cells were treated with 5 μM NaAsO_2_ or As_2_O_3_ for different times (0 to 72 h). Cell cycle distribution was detected by flow cytometry using PI staining to allow the exclusion of dead cells and normalization (since at 72 h too many cells are already dead). For each sample, 10,000 cells were collected and analyzed. Data were obtained from four independent experiments. The data represent the percentage of NaAsO_2_-treated cells or As_2_O_3_-treated cells in each phase of the cell cycle.

Next, we evaluated the impact of NaAsO_2_ and As_2_O_3_ treatments on mitochondrial bioenergetics. We measured the mitochondrial membrane potential (Fig 2A–2C) of the P3HR1^+^ treated cells together with the ATP (Fig 2D) and NAD(P)H determination (Fig 2E). NaAsO_2_ induced drastic drops in ΔΨm and NAD(P)H depletion that may affect the signal transduction pathways since these drops are associated with almost total ATP depletion (Fig 2D), which may affect the ATP-dependent cellular processes.

In contrast to NaAsO_2_, As_2_O_3_ treatment appeared to be more discrete since the reductions in ΔΨm (Fig 2A), NAD(P)H depletion (Fig 2E) and ATP levels (Fig 2D) are quite moderate. ΔΨm and ATP obviously stayed at levels where they could still sustain all phenomena linked to mitochondrial bioenergetics and mechanisms driven by ATP, i.e., some autophagic processes. The pan-caspase inhibitor QVD-OPH only affected the NaAsO_2_ treatment, with partial modulation of ΔΨm, whereas caspase inhibitors did not impact the ΔΨm value in response to As_2_O_3_ treatment (Fig 2B).

These results suggest that the treatment with each compound has a direct impact on the mitochondrial compartment. It cannot be excluded that the partial reduction in the ΔΨm drop could result from participation of the caspases (i.e., caspase-3 and caspase-9) in the action of NaAsO_2_. Treatment of the cells with the autophagy inhibitor 3-MA did not change anything regarding the modulation of the ΔΨm differentially induced by either As_2_O_3_ or NaAsO_2_ in the P3HR1^+^ cell line (Fig 2C).

### • As_2_O_3_ and NaAsO_2_ modify the redox state and inhibit the proliferation of P3HR1+ cells

**Fig 3:**
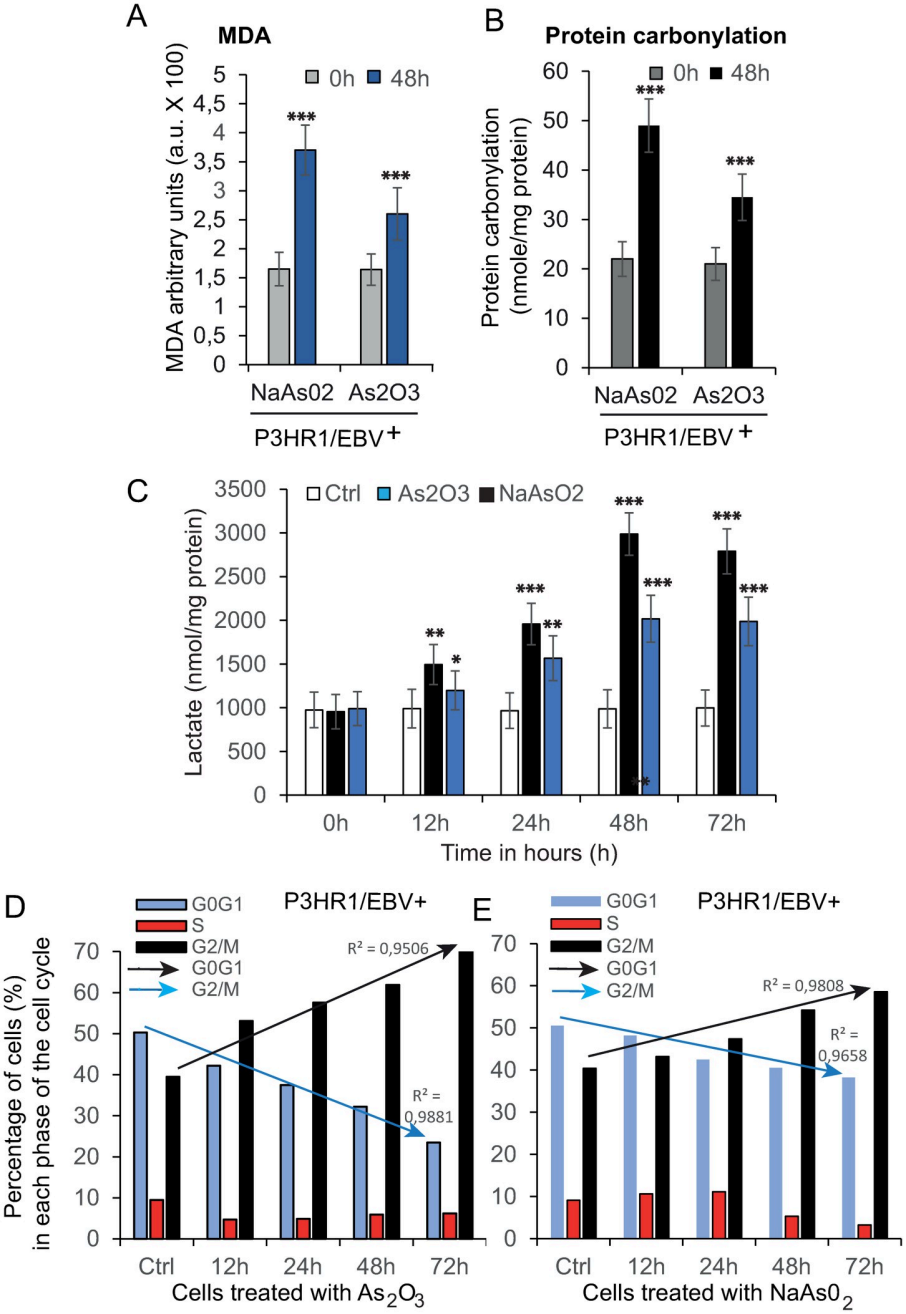
Effects of NaAsO_2_ and As_2_O_3_ on the mitochondrial bioenergetic properties of the P3HR1^+^ (EBV^+^) cells. A—Differential effects of 5 μM NaAsO_2_ and As_2_O_3_ on the mitochondrial membrane potential as measured by DiOC_6_(3) fluorescence measurements. B and C—Modulation of the effects of 5 μM NaAsO_2_ and As_2_O_3_ on the mitochondrial membrane potential when challenged by 10 μM pan-caspase inhibitor QVD-OPH (B) or by 5 μM autophagy inhibitor 3-methyladenine (C). D–Measurement of ATP level when the P3HR1 (EBV^+^) cells are treated with NaAsO_2_ and As_2_O_3_.

In order to test the potential toxic effects of As_2_O_3_ or NaAsO_2_ revealed by the loss of viability of P3HR1^+^ cells, we examined both lipid peroxidation (Fig 3A) and protein carbonylation (Fig 3B) induced upon treatments. As_2_O_3_ and NaAsO_2_ similarly affected protein carbonylation and lipid peroxidation. The similarity of the response with the two drugs could be due to the fact that over a certain time of exposure and over a certain threshold of concentration the maximum state of lipid peroxidation and of protein carbonylation is reached.

Nevertheless, such an appearance of peroxidized lipids is usually linked to a level of oxidative stress that may drive the onset of autophagy, mitophagy and aerobic glycolysis, resulting in the local production of high-energy mitochondrial fuels (such as L-lactate, ketone bodies and glutamine). Measurement of lactate production (Fig 3C) confirmed that these changes in lipid peroxidation and protein carbonylation are also associated with significant lactate production, suggesting changes in the cellular redox status. However, in this situation, the As_2_O_3_ treatment is less aggressive than the NaAsO_2_ treatment, the amount of lactate produced being greater with NaAsO_2_ than with As_2_O_3_, with an optimal level at 48 h. We then assessed the impact of As_2_O_3_ and NaAsO_2_ on the cell cycle. They both blocked the P3HR1+ cells in the G2/M phase of the cell cycle (Fig 3D and 3E). The blockade was more pronounced with NaAsO_2_ than with As_2_O_3_. Our experiments show that both species of arsenic delay the progression through each phase of the cell cycle and may induce cell death following G2/M arrest.

### • As_2_O_3_ and NaAsO_2_ generate ROS from the mitochondrial compartment and downregulate antioxidant defense

**Fig 4:**
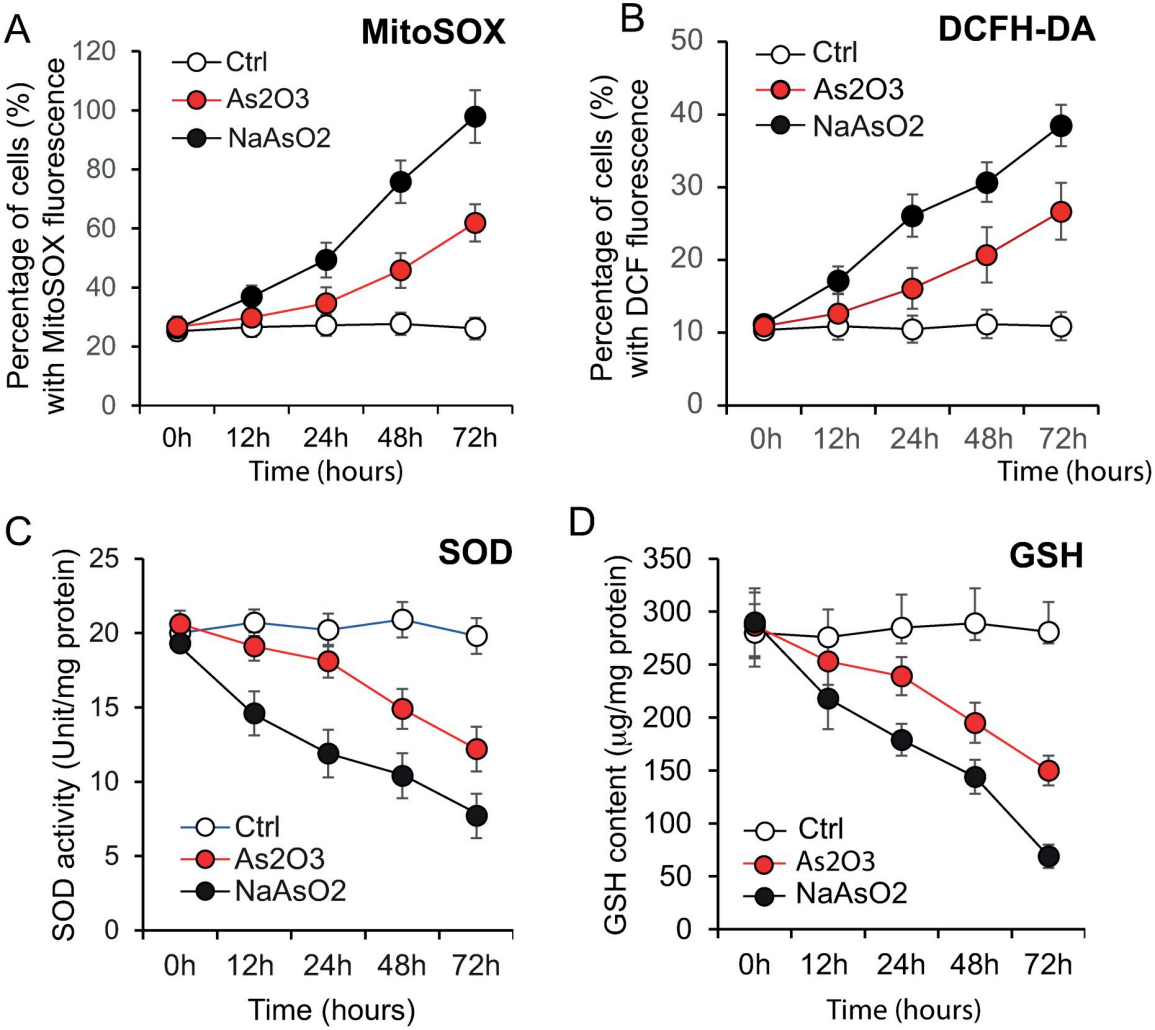
Effects of NaAsO_2_ and As_2_O_3_ on the level of ROS produced and possible down-regulation of the antioxidant defense [(SOD and GSH of the P3HR1 (EBV^+^) cell]). Cells were treated with 5 μM NaAsO_2_ or As_2_O_3_ for 0–72 h. The parameters of oxidative stress, including ROS, GSH and SOD, were measured. A -Intracellular ROS level was determined by using a fluorescent probe (DCFH-DA), and the fluorescent intensity of DCF was evaluated with an excitation wavelength at 488 nm and an emission wavelength of 530 ± 30 nm. PI staining was done concomitantly to ensure the exclusion of dead cells together with the light scattering properties. B–The superoxide anions that originate from mitochondrial respiratory chain dysfunction are measured with the MitoSOX red fluorescent probe. TO-PRO-3 iodide staining was performed concomitantly to ensure the exclusion of dead cells together with the light scattering properties. C—SOD activity was tested using a commercial SOD kit and normalized according to the protein concentration. D—The content of GSH was detected by flow cytometry analysis by using monobromobimane (mBrB). Data are given as the mean fluorescent value (F mean) and the ± SD is the standard deviation given along the curve (data from the flow cytometry data and the FlowJo software are the means of 6 independent experiments). Moreover, the background of the mBrB staining was deduced from the measurement with GSH depletion as indicated in the materials and methods. For (A) and (B), the data were taken from six independent experiments and expressed as mean ± S.D. One-way ANOVA analysis including the least significant difference (LSD-*t* test) multiple comparisons was performed to evaluate the statistically significant difference.

The clear effect of both As_2_O_3_ or NaAsO_2_ on the mitochondrial membrane potential (ΔΨm) is detected as a drop in fluorescence of the mitochondrial membrane potential-sensitive probe, i.e., DiOC_6_(3) related to mitochondrial bioenergetics defects [47, 48]. The drop in mitochondrial membrane potential, associated to the one of ATP strongly suggest that As_2_O_3_ or NaAsO_2_ contribute to ROS generation that we analyzed in Fig 4. As_2_O_3_- or NaAsO_2_-induced ROS production is linear, with increasing time of incubation of either As_2_O_3_ or NaAsO_2_ (Fig 4A and 4B). As observed throughout this study, NaAsO_2_-induced ROS production is more pronounced than As_2_O_3_-induced production.

More curiously, As_2_O_3_ and NaAsO_2_ also potently inhibit both superoxide dismutase (SOD) activity and glutathione level (GSH) at different intensities (Fig 4C and 4D).

Taken together, these data indicate that there is an increase in production of ROS, whether they are superoxide anions or hydrogen peroxide, which parallels the inhibition of antioxidant defenses represented by SOD activity or GSH cellular content.

### • Electron microcopy assessment of the differential effects of NaAsO_2_ and As_2_O_3_

**Fig 5:**
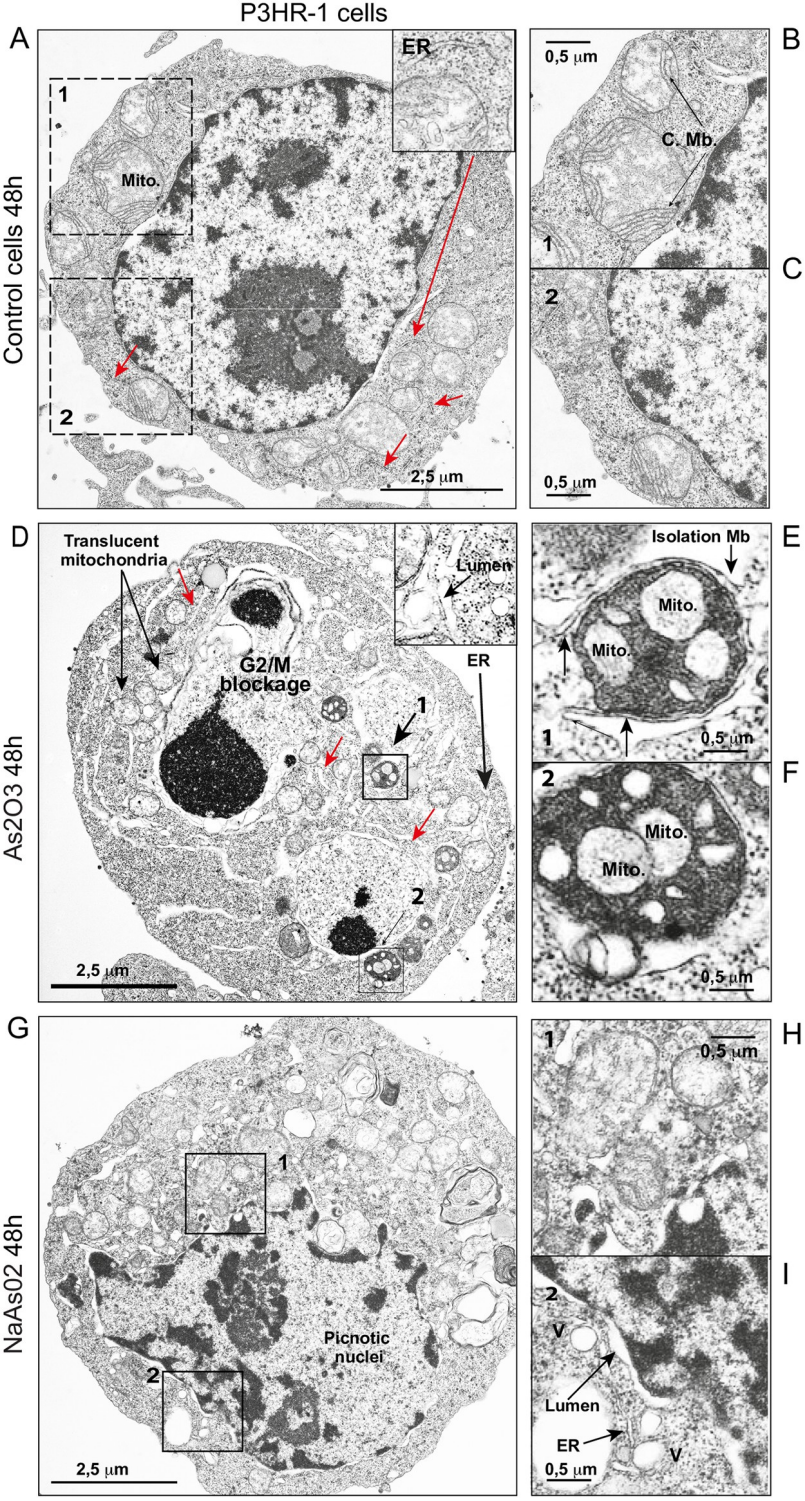
Flow cytometry analysis of acridine orange staining associated with LC3B detection. A—Image cytometry of the acidic compartment and LC3B staining of the autophagosomes of P3HR1+ cells treated with 5 μM As_2_O_3_ for 48 h. The measurements were done with an Amnis ImageStream100 (Amnis, Merck Millipore) imaging flow cytometer. The images presented are bright field (gray) and the red channel for the LysoTracker red fluorescence, the green channel for the LC3B antibody green fluorescence and the colocalization of green and red. B—Biparametric flow image cytometry analysis of EGFP-LC3 versus LysoTracker^™^ Deep Red of P3HR1+ cells treated with 5 μM As_2_O_3_ for 48 h as a proof of concept. C, D and E—Flow cytometric analysis of AO staining of PH3R-1 cells as described in materials and methods when treated or not (C—control) with either 5 μM arsenic trioxide (D) or 5 μM sodium arsenite (E) for 48 h. We obtained green fluorescence when the AO molecules were free or bound to DNA, whereas AO red fluorescence is emitted from an acidic environment where the molecules aggregate and form stacks (Stokes shift). The histograms of green and red fluorescence show the enhancement of the acidic compartment (red fluorescence), but also the death of cells, which usually lose their acidic vesicles and have a compacted DNA resulting in less green fluorescence (green fluorescence). F, G and H–Histogram presentation of the AO staining of control cells, 5 μM arsenic trioxide or 5 μM sodium arsenite (F) for different times. The same treatment in the presence of the pan-caspase inhibitor QVD-OPH (G) or the autophagy inhibitor 3-methyladenine (3-MA) (H).

Electron micrographs of P3HR1^+^ cells treated with 5 μM NaAsO_2_ or As_2_O_3_ for 48 h reveal a different morphology (Fig 5A–5I). The control cells exhibit a normal cytoplasm enclosing canonical round-shaped mitochondria with distinct cristae membranes (Fig 5B) and normal nuclei with dispersed DNA and clear nucleoli identification (Fig 5B and 5C). P3HR1^+^ cells treated with As_2_O_3_ exhibited the characteristics of cells blocked in G2/M in the cell cycle with distinct nuclei that have dense DNA (Fig 5D) and spectacular autophagosomes with double membranes that mainly enclose small mitochondria without any cristae membranes (Fig 5E and 5F). Their cytoplasm is disorganized, with a large ER network exhibiting a large lumen (Table 1, see ER swelling), the residual mitochondria being small and translucid, without cristae membranes (Fig 5D). The nuclear envelope is clearly detached and creates a large lumen within the cytoplasm. P3HR1^+^ cells treated with NaAsO_2_ are quite different (Fig 5G and 5H), since they exhibit picnotic nuclei with large lumen (Fig 5I) with condensed DNA (Fig 5G and 5I) in cytoplasm that contains a lot of vacuolar structures (Fig 5I) and condensed mitochondria mainly devoid of cristae membranes (some residual cristae membranes are punctate and round) (Fig 5H). Electron micrographs confirm a clear distinction between P3HR1^+^ cells treated with 5 μM NaAsO_2_ or As_2_O_3_ for 48 h: NaAsO_2_-treated cells are most likely dying (apoptosis and/or secondary necrosis) whereas As_2_O_3_-treated cells undergo a typical autophagic process, with a clear presence of mitophagic vesicles.

**Table 1:** Characteristics linked to the unfolded protein response.

Conditions	Ctrl	As_2_O_3_	NaAsO_2_
ER swelling (size in μm)	0.07± 0.02	**0.21 ± 0.09**	0.08 ± 0.03
Calcium increase (Fluo-4AM, a.u.)	135 ± 10	**690 ± 49**	175 ± 12
GRP78/BiP (a.u.)	0.44 ± 0.09	**0.8 ± 0.2**	0.45 ± 0.1
CHOP (a.u.)	0.38 ± 0.08	**1.5 ± 0.4**	0.4 ± 0.1

### • As_2_O_3_ induces an unfolded protein response, NaAsO_2_ does not!

Among the cellular characteristics observed in the electron micrographs is an ER swelling after As_2_O_3_ treatment (Fig 5 and Table 1). We further characterize an eventual ER stress in these cells. To do so, we analyzed the calcium content in P3HR1^+^ cells treated with either As_2_O_3_ or NaAsO_2_ for 48h. As_2_O_3_ clearly induced an increase in calcium (690 ± 49 versus control at 135 ± 10), whereas NaAsO_2_ did not (Table 1).

These events also correlated with upregulation of the transcription factor C/EBP homologous protein, (transcription factor) CHOP and a large increase in the 78-kDa glucose-regulated protein, GRP78 (Table 1), suggesting an unfolded protein response, a cellular stress response that could be related to an ER stress induced by As_2_O_3_. A detailed analysis of the cells treated with NaAsO_2_ revealed none of these manifestations, neither ER swelling nor marked calcium increase, and thus no CHOP or GPR78 activation (Table 1).

### • As_2_O_3_ and NaAsO_2_ induce respectively the formation or not of autophagosomes

We estimated LC3B mRNA as a function of time (Table 2) as a reporter of the probability of dysregulation of cellular steady-state autophagy. Clearly when cells are incubated with 5 μM NaAsO_2_ or 5 μM As_2_O_3_, these two compounds boost LC3B mRNA levels. The two compounds act differently, with As_2_O_3_ being more efficient than NaAsO_2_ in increasing LC3B mRNA relative expression, from 1 h post-treatment, with a peak at 48 h and a 3.5-fold increase in As_2_O_3_ treated cells compared to control.

**Table 2:** LC3B mRNA expression after treatment of P3HR1+ cells with NaAsO_2_ or As_2_O_3_ used at 5 μM. LC3B mRNA was quantified by RT-qPCR and normalized for housekeeping RPL13A (60S ribosomal protein L13a).

LC3B mRNA expression	NaAs0_2_	As_2_O_3_
Ctrl	1.65 ± 0.48	1.78 ± 0.51
1 h	2.56 ± 0.64	4.91 ± 0.91
8 h	2.72 ± 0.71	5.09 ± 0.98
24 h	2.74 ± 0.68	5.27 ± 1.04
48 h	2.71 ± 0.72	6.29 ± 1.29
72 h	2.39 ± 0.59	4.05 ± 0.89

**Fig 6:**
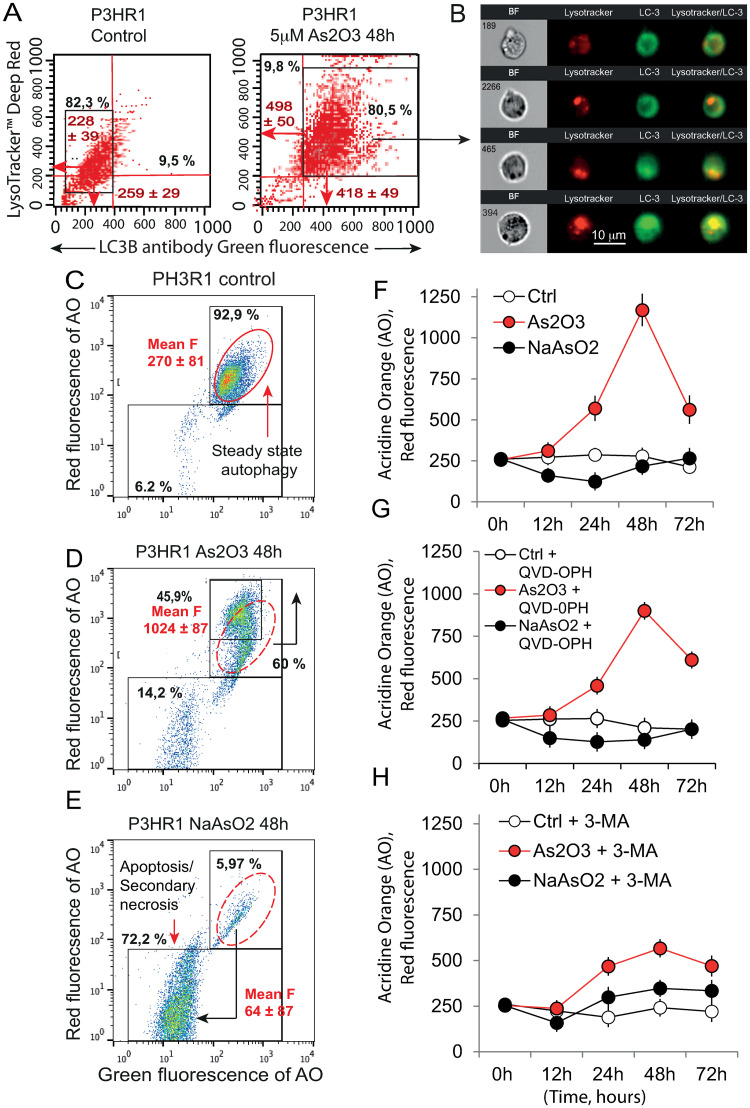
Electron microscopy of P3HR1 cells treated with either 5 μM NaAsO_2_ or As_2_O_3_ for 72 h. A—P3HR1 cells, control and highlighting of the mitochondrial compartment. With detailed mitochondria situated in the enlarged part of the cytoplasm designated as B and C. D—P3HR1 cells treated with sodium arsenite (NaAsO_2_ 5 μM, 72 h). The early phase of DNA condensation due to cell death is visible; the nuclei are undergoing pyknosis. E—Enlargement of the mitochondria in the cytoplasm that appear translucent and have lost their cristae membrane. Smaller mitochondria, resulting from mitochondrial fission, are also present. F—Enlargement of a portion of cytoplasm and nuclei allows the detection of a large lumen at the nuclear membrane that is characteristic of the cell death process. It can also be seen that the ER is slightly swollen and numerous vacuoles are present. G—P3HR1 cells treated with arsenic trioxide (As_2_O_3_ 5 μM, 72 h). The cells are clearly blocked in the cell cycle in G2/M (see Fig 4). The nuclei present marked condensation of the DNA. The mitochondria are numerous, small, translucent, without cristae membranes and widely condensed close to the nuclei. In H and I, autophagosomes. H—The enlarged picture shows an autophagosome with a well-defined isolation membrane (a double membrane that surrounds the content). Mitochondria are enclosed in the autophagosome. H—Enlargement of a portion of cytoplasm and nuclei allows the detection of a large lumen at the nuclear membrane that is characteristic of the cell death process. The ER is slightly swollen and numerous vacuoles are present. I—The enlarged picture shows an autophagosome with a well-defined isolation membrane (a double membrane that surrounds the content). Mitochondria are enclosed in the autophagosome. Abbreviations used in the pictures: Mb, Cristae membrane; ER, endoplasmic reticulum; Isolation Mb; Isolation Membrane, Mito., mitochondria; V, vacuole. The sizes in μm of the diverse intracellular organelles are indicated by the bars (^__^, in black).

In image cytometry analysis we focused on double staining of P3HR1^+^ cells with LysoTracker Deep Red combined with an LC3B immunoassay (Fig 6). The relocation of LC3B puncta to autophagosome membranes associated with an increase in lysosomal content is well documented as a hallmark of autophagy [49]. So, we used the ImageStream system on selected double-stained cells to analyze the colocalization of LysoTracker Red and LC3B green immunostaining, which would reflect the formation of autolysosomes (Fig 6A). Staining of P3HR1^+^ cells by both the LysoTracker Deep Red and the LC3B green immunofluorescence is evidenced by LysoTracker/LC3B colocalization into 85% of the As_2_O_3_ treated cells that differed clearly from control cells (Fig 6B, Table 3). As_2_O_3_ induced therefore the formation of autolysosomes in P3HR1+ cells, whereas NaAsO_2_ did not affect them significantly.

**Table 3:** Interaction of LC3B puncta with the acidic compartment stained with LysoTracker Red as a function of the treatment with NaAsO_2_ or As_2_O_3_ after 48-h hour incubation. The percentage (%) of cells that exhibit both LC3B green fluorescence and LysoTracker Red fluorescence is given, as the percentage (%) of cells that exhibit strict colocalization, meaning that LC3B puncta colocalized with the compartment positive to LysoTracker Red (LysoT).

Treatment	5 μM NaAsO_2_, 48 h	5 μM As_2_O_3_, 48 h
Cells which have LysoTracker Red and LC3B fluorescence	4.95 ± 0.97%	87.9 ± 3.6%
Cells with total colocalization of LC3B and LysoTracker Red	1.95 ± 0.51%	85.5 ± 7.1%
Bright detail similarity* LC3B/LysoT	≤ 1 for 97% of cells	≥ 1 for 86% of cells

*The similarity is high and significant when evaluated as being ≥ 1

To investigate further the induction of autophagy by As_2_O_3_ and not NaAsO_2_, we used acridine orange (AO) staining to analyze the formation of acidic vesicular organelles (AVO) compartment induced by both treatments, a feature of cells engaged in autophagy as described previously [43, 45, 50]. Flow cytometry measurements were used to monitor the formation of AVO by AO red together with the amount of cell death (when the green fluorescence also decreases due to DNA condensation). In cells treated with 5 μM NaAsO_2_ or As_2_O_3_ for 48 h (Fig 6C–6H), AO staining was first described as a validated proof of concept (Fig 6C–6E), with steady-state AVO measured by mean red fluorescence at 270 ± 81 nm for 92.9% of the cells (Fig 6C), whereas As_2_O_3_-treated cells (Fig 6D) exhibit a clear increase in red fluorescence to the mean value of 1024 ± 87, which mainly concerns 45.9% of the cells, together with the appearance of a low red and low green population, corresponding to dead cells (14.2%). NaAsO_2_ treatment does not modify the AVO compartment, but generates in the same conditions strict cell death for 72.2% of the cells (Fig 6E). A detailed time-course analysis from 0 h to 72 h (Fig 6F–6H) was used to monitor the modulation of the AVO formation, with a peak at 48 h for As_2_O_3_, whereas NaAsO_2_ did not seem to increase AVO formation, suggesting therefore that only the As_2_O_3_ treatment induces an autophagic response.

Then, we tested whether the pan-caspase inhibitor, QVD-OPH, or the inhibitor of autophagy 3-MA could modulate the effects of As_2_O_3_ on the AVO compartment (Fig 6F–6H). From Table 4, it can be seen that caspase-8 is clearly activated, in comparison to the mild activation of caspase-3 and 9. Caspase-1 is not activated in this context. In contrast, upon 48 h NaAsO_2_ treatment, caspase-8 is highly activated as are caspase-3 and -9, which are “executive” caspases acting downstream of the mitochondria, in line with dominance of apoptosis over autophagy. As a result, if there is a contribution of diverse caspases to the induction of As_2_O_3_-induced AVO, it is marginal (Fig 6G), whereas 3-MA downregulated As_2_O_3_-induced AO increase, (Fig 6F and 6H), demonstrating that AVO formation induced by As_2_O_3_ originates from the autophagy induction.

**Table 4:** Caspase activities of P3HR1 cells treated with either 5 μM NaAsO_2_ or 5μM As_2_O_3_ for 48 h.

Treatment for 48 h	5 μM As_2_0_3_		5 μM NaAsO_2_	
	Percent cells	F mean	Percent cells*	F mean
Caspase-1	92 ± 5	101 ± 9	106 ± 11	106 ± 11
Caspase-8	67 ± 5	399 ± 39	59 ± 7	709 ± 42
Caspase-3	90 ± 3	250 ± 54	91.4 ± 3	761 ± 61
Caspase-9	91 ± 4	248 ± 57	90.9 ± 4	756 ± 63

**Fig 7:**
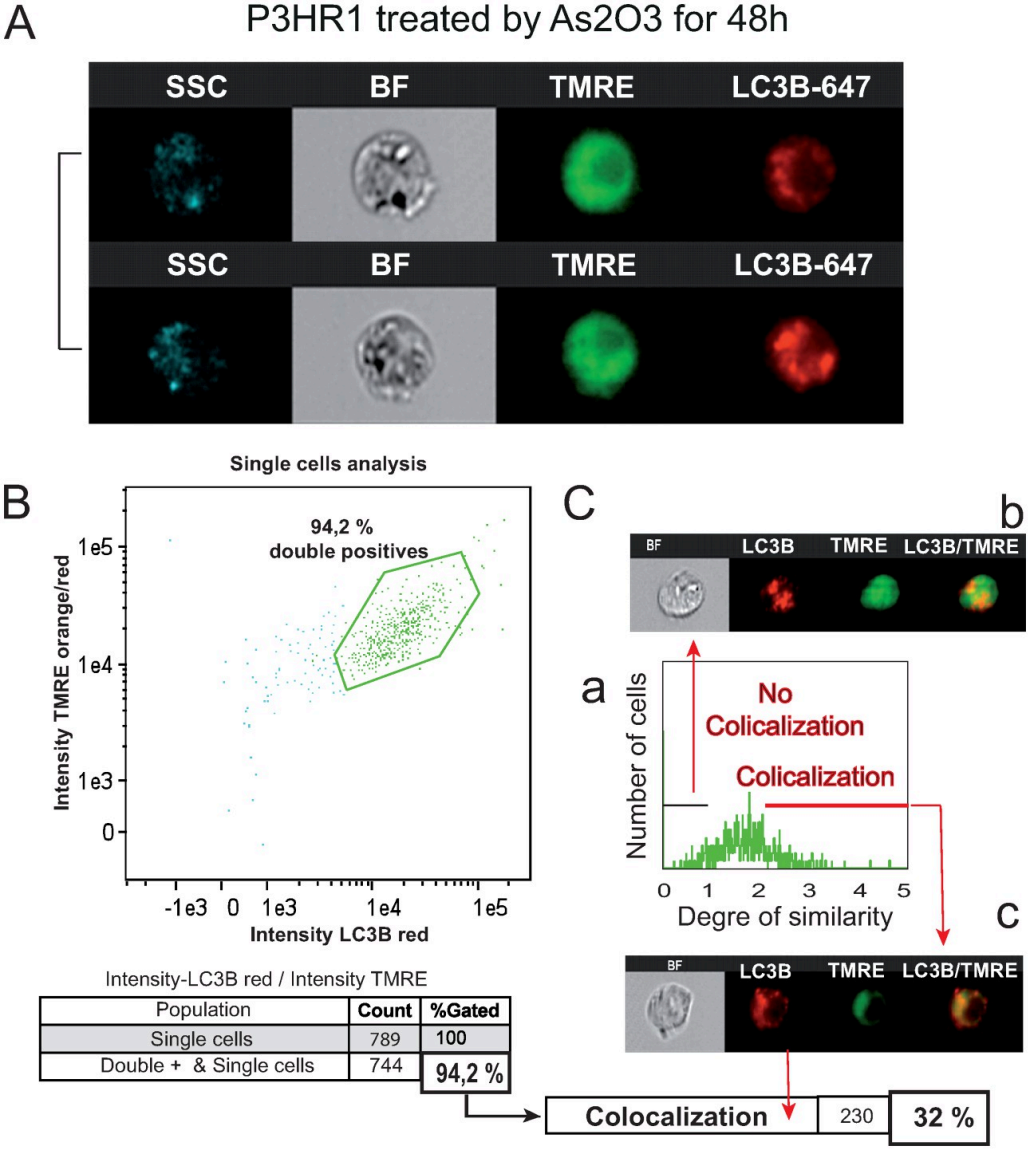
Image cytometry (Amnis) of the double staining of LC3B cells treated with 5 mM ATO for 48h stained with TMRE and LC3B-647. A—Example of single cell analysis for two cells (2) obtained after 48h incubation with 5 mM ATO and double staining by 40 nm TMRE (orange/red fluorescence) and LC3B-647 (far red fluorescence) taken in false green fluorescence. B—Biparametric histogram of Green (false color) of TMRE orange/red and, LC3B-647 fluorescence. A double stained population is highlighted representing 94,2% percent (744) of the whole population analysed (789 cells at the total). C—Similarity analysis for correlation between TMRE fluorescence and LC3B-647 fluorescence. The analysis of the colocalization of the TMRE and LC3B-647 has been performed by using the Similarity Score included in IDEAS 6.0 software™ (Amnis). This score, a log-transformed Pearson’s correlation coefficient between the pixels of two image pairs, provides a measure of the degree of co-localization by measuring the pixel intensity correlation between the TMRE and LC3B-647 images. Analysis performed on the 744 cells that exhibit a double staining as seen in (B). (a) A histogram giving the Gaussian repartition of the cells in term of similarity indicating or not the perfect colocalization of the two probes. (b) Cells with no or to little colocalization (c) Cells with almost full colocalization that have malfunctioning mitochondria enclosed in autophagosomes.

As electron microscope images from Fig 5 suggest that As_2_O_3_ treatment could induce mitophagy, we assessed the intracellular distribution of the red fluorescence of TMRE (to label mitochondria, presented in false green color in Fig 7) and 647 nm fluorophore-linked to LC3B by flow cytometry imaging (Fig 7A). The biparametric histogram allowed us to detect cells being double stained (Fig 7B), representing 94,2% of the cells treated with ATO for 48h. The LC3B-647 staining appeared heterogeneous but discrete, while TMRE fluorescence was much more widespread (Fig 7A). So, we performed a similarity analysis on the Amnis system to see whether the chosen cells (those presenting double staining) exhibited a similar colocalization of LC3B-647 and TMRE green fluorescence (Fig 7C). The histogram presented in Fig 7C shows that similarity scores under 1 correspond to a non-colocalized population (Fig 7C, b), while similarity scores over 2 concerned a significant colocalization signal observed in cells (Fig 7C, c). This population accounted for 32% of the total double-stained population cells after 48 h treatment of As_2_O_3_, while they were barely detectable in non-treated cells (data not shown). These results clearly confirmed the data form electron microscopy (Fig 5E and 5F) where mitochondria almost devoid of cristae are observed within autophagosomal structures. Then demonstrating that a quite important level of mitophagy could be observed when cells are treated with ATO.

### • Role of the Epstein-Barr virus in the metabolic characteristics of cells when treated with either NaAsO_2_ or As_2_O_3_?

Since it has been previously shown that arsenite reactivates EBV in P3HR1 cells [51], we were interested to test whether EBV could affect the cellular response to NaAsO_2_ or As_2_O_3_ treatment by using Ramos-1 cells devoid of EBV infection (EBV^-^ cells), as previously used [51].

While an As_2_O_3_ treatment impacts similarly the increase in peroxidized lipids and protein carbonylation in the two cell types (Fig 3 and S2A Fig), Ramos-1 cells were more sensitive to this treatment in terms of viability (S1A Fig). Considering cellular bioenergetics, i.e., the mitochondrial membrane potential or the level of reduction of NAD(P)H, it appears that there is mostly no difference between EBV^+^ (Fig 2) and EBV^-^ cells (S2A and S2B Fig) in terms of effects on mitochondrial bioenergetics (Fig 2) upon both NaAsO_2_ and As_2_O_3_ treatments. Nevertheless, the clear difference between the actions of NaAsO_2_ and As_2_O_3_ is still present, with NaAsO_2_ provoking a huge drop in ΔΨm and reduction of NAD(P)H to give rise to NAD(P)^+^, whereas As_2_O_3_ induces the same events but to a much lesser extent. Regarding the production of ROS, i.e., superoxide anions and hydroperoxide (S2C and S2D Fig, respectively), and/or the inhibition of the antioxidant defense system, via estimation of superoxide dismutase activity or determination of GSH content (S2E and S2F Fig, respectively), it appears that there is no change depending on the presence or absence of EBV. In any case, the production of ROS is greatly increased with the NaAsO_2_ treatment, whereas there is only a slight increase of ROS when cells are treated with As_2_O_3_.

**Fig 8:**
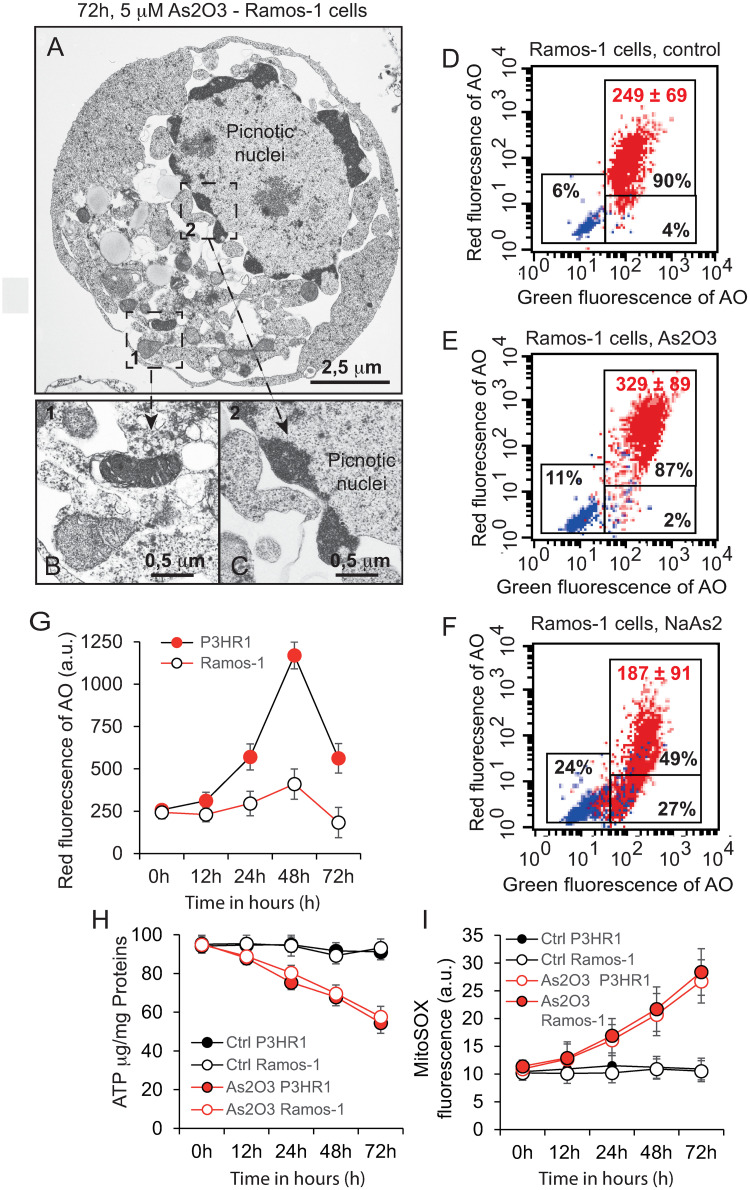
Electron microscopy of Ramos-1 cells and detailed information about their autophagic and cell death status associated with bioenergetic characteristics. A, B, C Electron microscopic images of Ramos-1 (R-1, EBV^-^) cells treated with either 5 μM NaAsO_2_ or As_2_O_3_ for 72 h. B and C are enlargements of A in order to focus on mitochondria in B and on the picnotic appearance of the nuclei (with condensed DNA) in C. D-G, Fine analysis of acridine orange staining of the cells in different conditions: A detailed flow cytometry analysis is given for control cell staining (D), cells treated 48 h with 5 μM As_2_O_3_ (E) or with 5 μM NaAsO_2_ (F). G—Comparison between P3HR1 (in red circles) and Ramos-1 (empty circles) cells treated with 5 μM As_2_O_3_ for different times. H and I, bioenergetic information about Ramos-1 cells treated with As_2_O_3_ for different times.

In line with the decreased viability observed in S1A Fig, electron micrographs of the Ramos-1 EBV^-^ cells treated for 72 h with As_2_O_3_ (Fig 8A–8C) clearly differ from those of P3HR1^+^ cells (Fig 6D), since instead of a “canonical” appearance of autophagic cells, they exhibit a clear apoptotic phenotype (or mainly of early secondary necrosis following apoptosis initiation). Their nuclei appeared clearly picnotic, with the DNA packed at the nuclear periphery (Fig 8C). Also, the nuclear membrane was dissociated from the nuclei, the cytoplasm was disorganized, with electron-dense small mitochondria (Fig 8B), a lot of small empty vacuoles and round-shaped vesicles of fatty acid accumulation. So, apparently in the absence of EBV the cells treated with As_2_O_3_ have a post-apoptosis phenotype that is quite similar to that of the P3HR1^+^ cells treated with NaAsO_2_, combined with a less resistance to cell death. Consequently, we decided to compare the quantity of acidic vesicles stainable with AO (Fig 8D–8G) and, to our surprise, there was a clear-cut difference between EBV^+^ and EBV^-^ cells treated with As_2_O_3_. As can be seen on the flow cytometry histograms of AO staining (Fig 8D–8F), dysregulation of the AVO compartment was minimal when cells were treated with As_2_O_3_ (with a mean red fluorescence of 329 ± 89 compared to the control of 249 ± 69) (Fig 8D and 8E), whereas NaAsO_2_ treatment induced a slight decrease of AVOs and promoted a clear apoptotic/necrotic cell death as observed in the same conditions for the P3HR1^+^ cells (Fig 6). This is evident when the two types of cells were subjected to a time-course detection of the red fluorescence of AO (Fig 8G). Overall, these results suggest that the boost of autophagy observed in As_2_O_3_ treated P3HR1^+^ cells could be due to the presence of EBV in these cells, since Ramos/EBV^-^ autophagy was not activated upon As_2_O_3_ and this boost of autophagy could delay the ATO-induced cell death, which is specific to P3HR1^+^ cells.

## Discussion

Arsenic is a common metalloid that occurs naturally in the environment (or due to industrial processes), and exists in inorganic and organic forms [2]. NaAsO_2_ and As_2_O_3_ are the two major forms of inorganic trivalent arsenic. NaAsO_2_ is a well-documented carcinogen, while As_2_O_3_ appears to be not only a poison but also an effective therapeutic tool in the treatment of APL and some solid tumors [22, 52]. Numerous studies have shown that inorganic trivalent arsenic induces paradoxical effects in the target cells [53, 54], but the detailed mechanisms underlying the different effects of NaAsO_2_ and As_2_O_3_ are still elusive. They involve metabolic sequences strictly linked to ROS production [55–59] via the mitochondrial compartment, and involve autophagy and/or apoptosis both of which are known to be linked by the same triggers [60].

### • As_2_O_3_ and NaAsO_2_ affect P3HR1+ cell viability and differential cell volume changes

We tried to distinguish the effects of two arsenical compounds, As_2_O_3_ and NaAsO_2_, on the P3HR1^+^ cell line, which is derived from an EBV^+^ Burkitt lymphoma.

As a first attempt to characterize the impact of these two arsenicals on the P3HR1+ cell line, we studied changes in viability and changes in cell volume over time. The action of NaAsO_2_ and As_2_O_3_ on the flow cytometric light-scattering properties of P3HR1^+^ (Fig 1A) clearly shows that NaAsO_2_ induces changes in light scattering along with a reduction in FSC and an increase in SSC that is characteristic of the development of apoptotic cell death (Fig 1A, central panel), and that As_2_O_3_ induces a huge cellular swelling reminiscent of autophagy (Fig 1A, right panel). The flow cytometry data collected from 0 to 72 h in terms of FSC (“linearly related to cell size”) (Fig 1B) and SSC (cellular granularity) (Fig 1C) also correlate with an alternative measurement, i.e., the Coulter volume measurements (Fig 1D), and showed that whereas 5 μM NaAsO_2_ induced cell shrinkage associated with increased granularity, As_2_O_3_ provoked cellular swelling with quite fixed granularity.

The fine measurement of cellular viability by different sized DNA markers YOPRO-1 and PI that do not cross intact membranes but penetrate depending on the level of permeability, allows detection of apoptotic cells that are slightly permeable to YOPRO-1 but not to PI (YOPRO-1^+^PI^-^, apoptotic cells) and, later, the cells undergoing secondary necrosis become YOPRO-1 positive and PI positive (YOPRO-1^+^/PI^+^). The two arsenicals induce a loss of cellular viability, with 55.9% of cells permeable to As_2_O_3_ (30.5% of YOPRO-1^+^PI^-^ cells and 25.4% of YOPRO-1^+^/PI^+^ cells), whereas NaAsO_2_ induces 97.1% of totally permeable cells that are YOPRO-1^+^/PI^+^ (Fig 1E). The population of permeable cells under As_2_O_3_ treatment is only modestly reduced (5.2%) in the presence of the pan-caspase inhibitor QVD-OPH (Fig 1F), whereas there is a huge effect on NaAsO_2_-treated cells with almost total restoration of cellular viability (Fig 1F).

Also, P3HR1^+^ treatment with As_2_O_3_ and the autophagy inhibitor 3-MA drastically changed the ratio of induced permeabilization by abolishing the cells presenting a YOPRO-1^+^ and PI^—^state, to the benefit of YOPRO-1^+^/PI^+^ cells (i.e., dead/necrotic cells) (Fig 1G) compared to the control (Fig 1E). In contrast, 3-MA treatment did not significantly change the death recorded under NaAsO_2_ treatment since these cells are killed by caspase-8, caspase-3 and caspase-9 activation (Table 4) and not by any significant autophagic process.

As a first conclusion, the processes provoked by 5 μM As_2_O_3_ or NaAsO_2_ treatment for 48 h totally differ in nature. As_2_O_3_ induces marked autophagy, whereas NaAsO_2_ leads to cell death reminiscent of apoptosis and secondary necrosis. These results correlate well with the literature data suggesting that sodium arsenic induces apoptosis and then secondary necrosis while arsenic trioxide (As_2_O_3_) mainly triggers autophagy [61–63].

### • The two arsenical compounds impact cellular bioenergetics by differential stress induction

As_2_O_3_ binds to protein thiol groups, disrupting normal protein folding and function [64, 65]. It has long been known that the arsenicals NaAsO_2_ and As_2_O_3_ affect mitochondrial bioenergetics [48, 66, 67–69]. Also, sodium arsenite (NaAsO_2_) acts through the mitochondrial compartment. As an example, *in vitro* exposure of primary hepatocytes to an environmentally relevant level of NaAsO_2_ (μM range) results in increased oxidative stress that appears to arise from changes in the expression and activity of respiratory Complex I of the mitochondrial proton circuit [70]. For short incubation (< 24 h or less), ATO (As_2_O_3_) induces an apoptotic-like pathway directly acting on mitochondrial permeability transit.

Our findings are consistent with these previous reports in which mitochondrial ROS production is considered to be an important mechanism in As_2_O_3_-induced death [71, 72].

As the two drugs induce distinct levels of response on mitochondrial membrane potential, NAD(P)H reduction and decreased ATP production (Fig 2), they also induce differentiated activation of caspases (Table 4). In addition, NaAsO_2_ acts, at least partially, through interaction with the voltage-dependent anion channel. This being associated with opening of the permeability transition pore and cytochrome *c* release [73], this leads to so-called apoptotic cell death.

These events, strictly linked to mitochondrial behavior, take place in a context where the two compounds induce different levels of lipid peroxidation, protein carbonylation and an increase in lactate production (Fig 3). Induction of these three events was less pronounced with sodium arsenite (NaAsO_2_) than with arsenite (As_2_O_3_) (Fig 3), meaning that the level of cellular stress induced by the two compounds differs and the oxidative stress is greater with NaAsO_2_ treatment.

### • The cell cycle is also differentially affected by the two compounds in a context of deleterious ROS production

The mechanism of growth inhibition by arsenite has been studied in detail [74] and involves delayed progression in all cell cycle phases with apoptosis ensuing at the G2/M phase. Among the possible explanations, the fact that arsenite binds to protein thiol groups, disrupting normal protein folding and function [64, 65], is the most reliable. Our results confirm this hypothesis, since As_2_O_3_ treatment of P3HR1^+^ cells induce a blockade in the G2/M phase of the cell cycle (Fig 3D).

### • As_2_O_3_ also induces a more potent unfolded protein response via the ER compartment than NaAsO_2_

In this study, we compared the influence of these two compounds on cell proliferation, cell cycle distribution (Fig 3D and 3E), oxidative stress (Fig 4A and 4B) and antioxidant defense modulation (Fig 4C and 4D), genetic damage, and apoptosis, which were directly or indirectly associated with their paradoxical effects, in cells. Our results demonstrate that both NaAsO_2_ and As_2_O_3_ induce oxidative stress with subsequent DNA damage, thereby leading to cell cycle arrest and cell death (Fig 3). NaAsO_2_ was more effective than As_2_O_2_ in inducing these observed effects (oxidative level, G2M blockade and cell death).

### • The cell death mechanisms at work with As_2_O_3_ relate to increased autophagy, whereas NaAsO_2_ provokes mitochondrially induced apoptosis

Careful examination of the electron micrographs of P3HR1+ cells treated with As_2_O_3_ and NaAsO_2_ (Fig 5) showed that As_2_O_3_ promotes an autophagic process, including mitophagy as assessed by image cytometry (Fig 7) and the electron microscopy images, where deleterious mitochondria are enclosed in a double membrane (Fig 5E and 5F) and in a context where the cells are blocked in the G2/M state of the cell cycle (Fig 5D). In contrast, cells treated with NaAsO_2_ exhibit swollen mitochondria with translucent cristae (Fig 5H), with cytoplasm that exhibits swollen ER which looks empty (Fig 5I). The cells appear to have a destructured cytoplasm with picnotic nuclei (Fig 5G) more reminiscent of classical mitochondrially induced apoptotic death.

The combination of images in flow analysis with LysoTracker^™^ Deep Red and LC3B Green fluorescent antibodies (Fig 6A and 6B) and AO flow cytometry analysis (Fig 6C–6H) as well as TMRE with LC3B (Fig 7) confirmed that As_2_O_3_ induces autophagy and mitophagy, whereas NaAsO_2_ induces potent cell death mechanisms in P3HR1^+^ cells. It is quite interesting to note that the autophagic events are only slightly diminished by the pan-caspase inhibitor, QDV-OPH, whereas the cells treated with 3 methyladenine (3-MA) exhibit marked reduction in autophagy in 48 h culture, which could lead to a marked protection of the cells from death.

### • EBV^-^ cell (i.e., Ramos cells) bioenergetics do not differ from EBV^+^ cell (P3HR1+ cells) bioenergetics but autophagy is not promoted upon As_2_O_3_ treatments

Whatever the measurements related to dysfunction of the mitochondrial compartment following the treatment of Ramos-1 cells with either As_2_O_3_ or NaAsO_2_, the main conclusions were similar. The actions of As_2_O_3_ and NaAsO_2_ on Ramos-1 cells are mainly distinguishable from each other by the intensity of their damage (S2 Fig), as for P3HR1^+^ cells (Fig 2). Effectively, As_2_O_3_ only mildly affects the mitochondrial membrane potential and NAD(P)H oxidoreduction, whereas NaAsO_2_ treatment induces a large drop in ΔΨm (S2A Fig and Fig 2A) as well as almost total reduction of NAD(P)H (Fig 2E and S2B Fig). The same general effects can be attributed to the levels of superoxide anions and hydroperoxide, with a clear domination of NaAsO_2_ over As_2_O_3_ (Fig 4A and 4B and S2C and S2D Fig), whereas the activity of SOD and GSH are more preserved with As_2_O_3_ than with NaAsO_2_ (Fig 4C and 4D and S2E and S2F Fig).

The absence of EBV (Ramos-1 cells) or the presence of EBV (P3HR1+ cells) could be the main factor that makes a difference in terms of autophagy when cells are treated with either As_2_O_3_ or NaAsO_2_. Indeed, Ramos-1 cells treated with As_2_O_3_ (Fig 8A) exhibit defective mitochondria and destabilized cytoplasm, but also have a picnotic appearance at the nucleus level which is more related to a general cell death process than to the autophagic picture detected in P3HR1^+^ cells (Fig 5D). Therefore, the absence of EBV could inhibit As_2_O_3_-induced autophagy. It is also clear that the ATP level (Fig 8H) and mitochondrial superoxide anion production (Fig 8I) are not key to the behavior of Ramos-1 and P3HR1^+^ cells.

## Conclusion

It has recently been demonstrated that As_2_O_3_ does not induce the EBV reactivation via ubiquitin-mediated degradation. The consequences of this are inhibition of EBV replication and induction of cell death in EBV^+^ cells, even if the nature of the death observed has not been characterized [75]. This is consistent with other reports that EBV genome loss and failure of lytic gene expression leads to a loss of malignancy phenotype and of cell viability in EBV^+^ Burkitt lymphoma cells [76–78]. It is currently thought that some cells in the lytic cycle show tumor cell growth and survival by furnishing cell growth factors and other signals. So, the drop in lytic gene expression after ATO exposure eradicates EBV genome replication and results in EBV-linked cell death [75], but clearly these events are secondary to the important autophagic processes depicted in the present work.

Autophagy is a cellular pathway which, under stress conditions, degrades and recycles nutrients, thus promoting cell survival. Autophagy also serves as a defense mechanism against viral infection, and many viruses take advantage of this mechanism to promote their replication. EBV encoded protein like BHRF1 protein, which is a BCL2 homolog, may target and influence mitochondria. In this context, BHRF1 expression modifies mitochondrial dynamics [79] and stimulates DNM1L/Drp-1-mediated mitochondrial fission. An interesting fact is that concomitantly BHRF1 has a pro-mitophagic effect [79], since it directly stimulates the general autophagic flux and more particularly mitophagy by interaction with BECN1/Beclin 1 [80].

**Fig 9:**
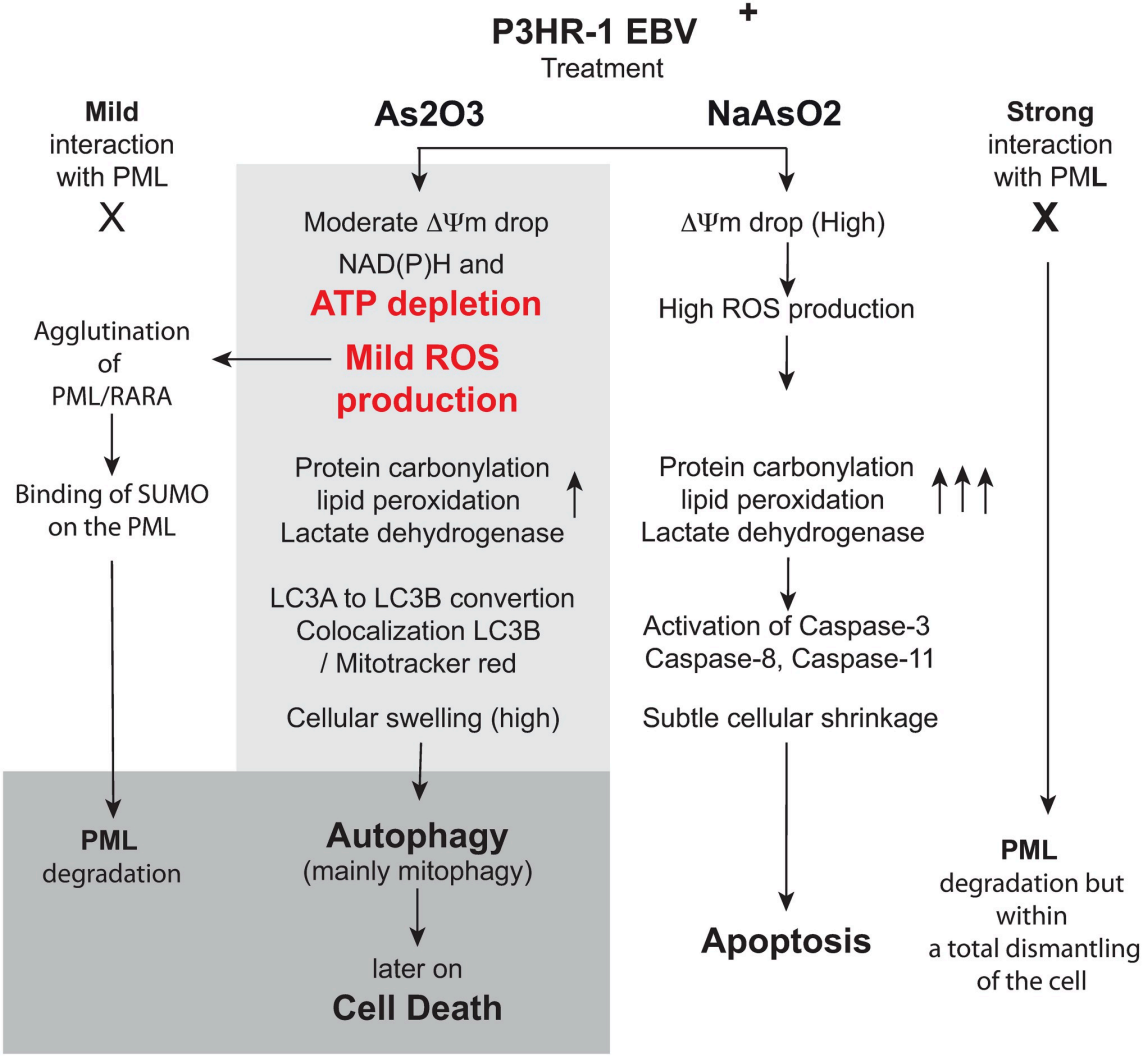
Schematic interpretation.

The situation is quite different when EBV^+^ P3HR1 cells are treated with As_2_O_3_, since autophagy and mitophagy are highly induced, which could delay cells from death, despite mitochondria alteration, decreased ΔΨm and reduced NAD(P)H, associated with increased ROS (Fig 9). EBV encodes several transcription factors like Rta [81] that activate autophagy via the extracellular signal-regulated kinase pathway. So, the control of autophagy by EBV to boost its proliferation and lytic cycle could be forced by As_2_O_3_ to a huge enhancement of autophagy, which could push the cells to a massive autophagy and consequently death at term (Fig 9).

So, as used in therapy in an EBV^+^ context, the combination of ATRA with ATO (As_2_O_3_) has changed APL from an extremely lethal disease to a highly curable condition, with chemotherapy-free approaches. This is quite clear since the two compounds act synergistically: ATRA-Era acts by inducing cell differentiation and inhibits cellular proliferation, and also acts on mitochondrial cardiolipin, leading to mitochondrial generation of ROS that provoke mitophagy [33], when ATO also induces global autophagy, which likely exceeds a threshold leading to cell death. This synergistic action specifically targets EBV^+^ cells, which exhibit stimulated autophagic fluxes compared to EBV^-^ cells.

## Supporting information

S1 Fig Ramos-1 cells, some characteristics, i.e., viability, lipid peroxidation and malondialdehyde production. A—Ramos-1 cells were treated with 5 mM of As_2_O_3_ or NaAsO_2_ for 72 h and stained with YOPRO-1/PI to determine their viability, that is the sum of YOPRO-1^+^/PI^-^, i.e., apoptotic cells + YOPRO-1^+^/PI^+^, necrotic cells (or “secondary necrosis”). All experiments have been repeated 7 time (n = 7). The bars represented the mean value of seven independent flow cytometric measurements (and is calculated from the mean value of what is called coefficient of variation at half-maximum or HCV from each experiment). Asterisks indicate statistically significant variation compared to the corresponding population in control cells, calculated using Student’s t-test (*P < 0.01, **P < 0.001). B—Malondialdehyde production at 0 h and 48 h for Ramos-1 cells treated with either 5 mM As_2_O_3_ or 5 mM NaAsO_2_ (n = 7). C—Protein carbonylation in Ramos-1 cells treated with 5 mM As_2_O_3_ or 5 mM NaAsO_2_ (n = 8).
(TIF)

S2 Fig Some bioenergetic characteristics of the Ramos-1 (EBV ^-^) cells treated with either 5 mM NaAsO_2_ or As_2_O_3_ for 72 h. A—Histogram representation of the mitochondrial membrane potential of Ramos-1 cells as detected with DiOC6(3) in flow cytometry (n = 12). B—Histogram representation of the NAD(P)H level detected by flow cytometry (n = 6). C—Superoxide anion production (n = 12). D—Hydroperoxide production (n = 12). E—Superoxide dismutase activity (n = 6). F—Glutathione synthase activity.
(TIF)

S1 File (XLSX)

S2 File (XLSX)

S3 File (XLSX)

S4 File (XLSX)

S5 File (XLSX)

S6 File (XLSX)
