# Supplementary figures and images for: Sodium arsenite and arsenic trioxide differently affect the oxidative stress of lymphoblastoid cells: An intricate crosstalk between mitochondria, autophagy and cell death
Source: PLoS One. 2024 May 10;19(5):e0302701. doi: 10.1371/journal.pone.0302701 (PMC11086853; doi:10.1371/journal.pone.0302701)

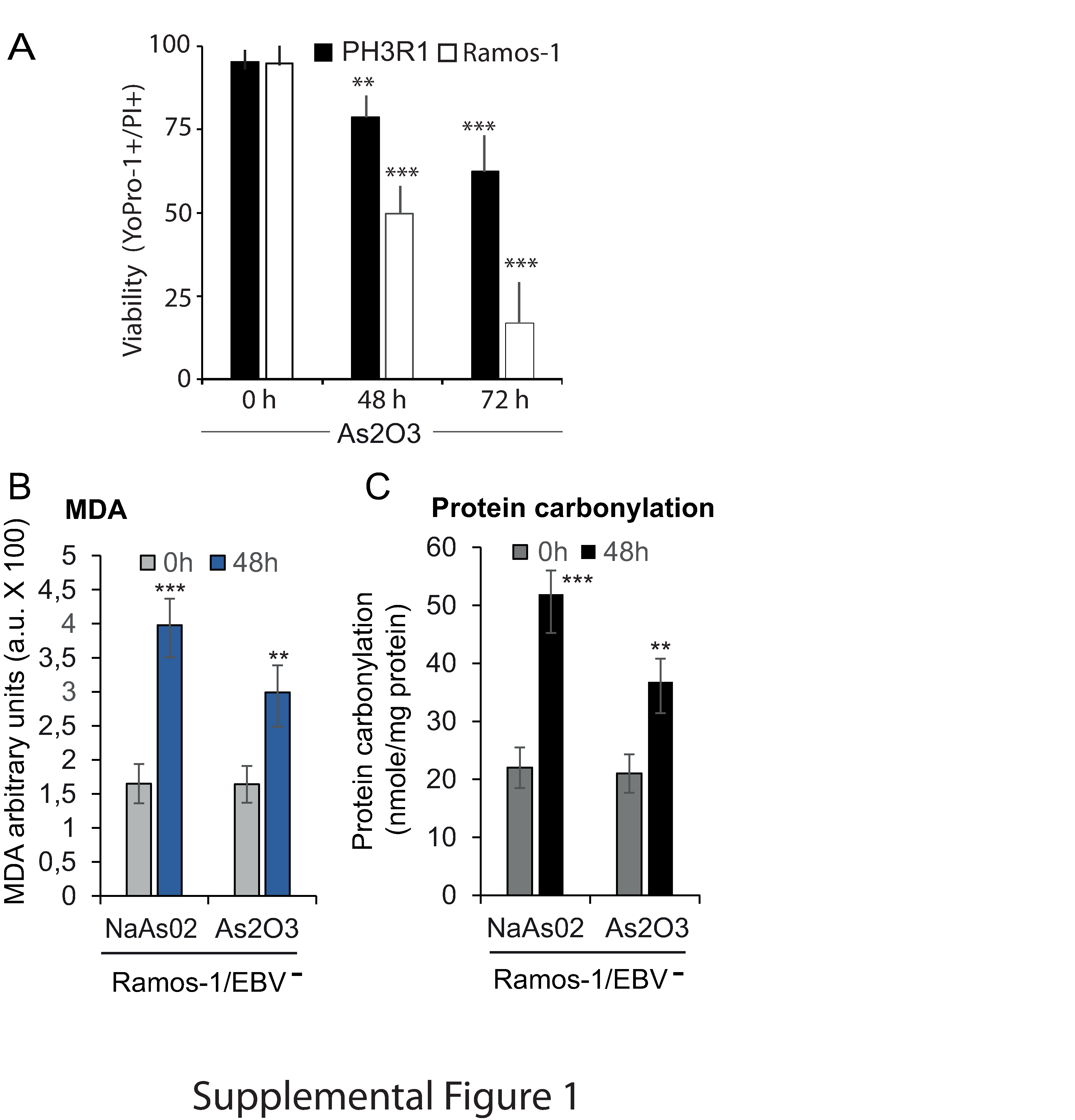

Supplement: S1 Fig — A—Ramos-1 cells were treated with 5 mM of As2O3 or NaAsO2 for 72 h and stained with YOPRO-1/PI to determine their viability, that is the sum of YOPRO-1+/PI-, i.e., apoptotic cells + YOPRO-1+/PI+, necrotic cells (or “secondary necrosis”). All experiments have been repeated 7 time (n = 7). The bars represented the mean value of seven independent flow cytometric measurements (and is calculated from the mean value of what is called coefficient of variation at half-maximum or HCV from each experiment). Asterisks indicate statistically significant variation compared to the corresponding population in control cells, calculated using Student’s t-test (*P < 0.01, **P < 0.001). B—Malondialdehyde production at 0 h and 48 h for Ramos-1 cells treated with either 5 mM As2O3 or 5 mM NaAsO2 (n = 7). C—Protein carbonylation in Ramos-1 cells treated with 5 mM As2O3 or 5 mM NaAsO2 (n = 8). (TIF) [file pone.0302701.s001.tif]

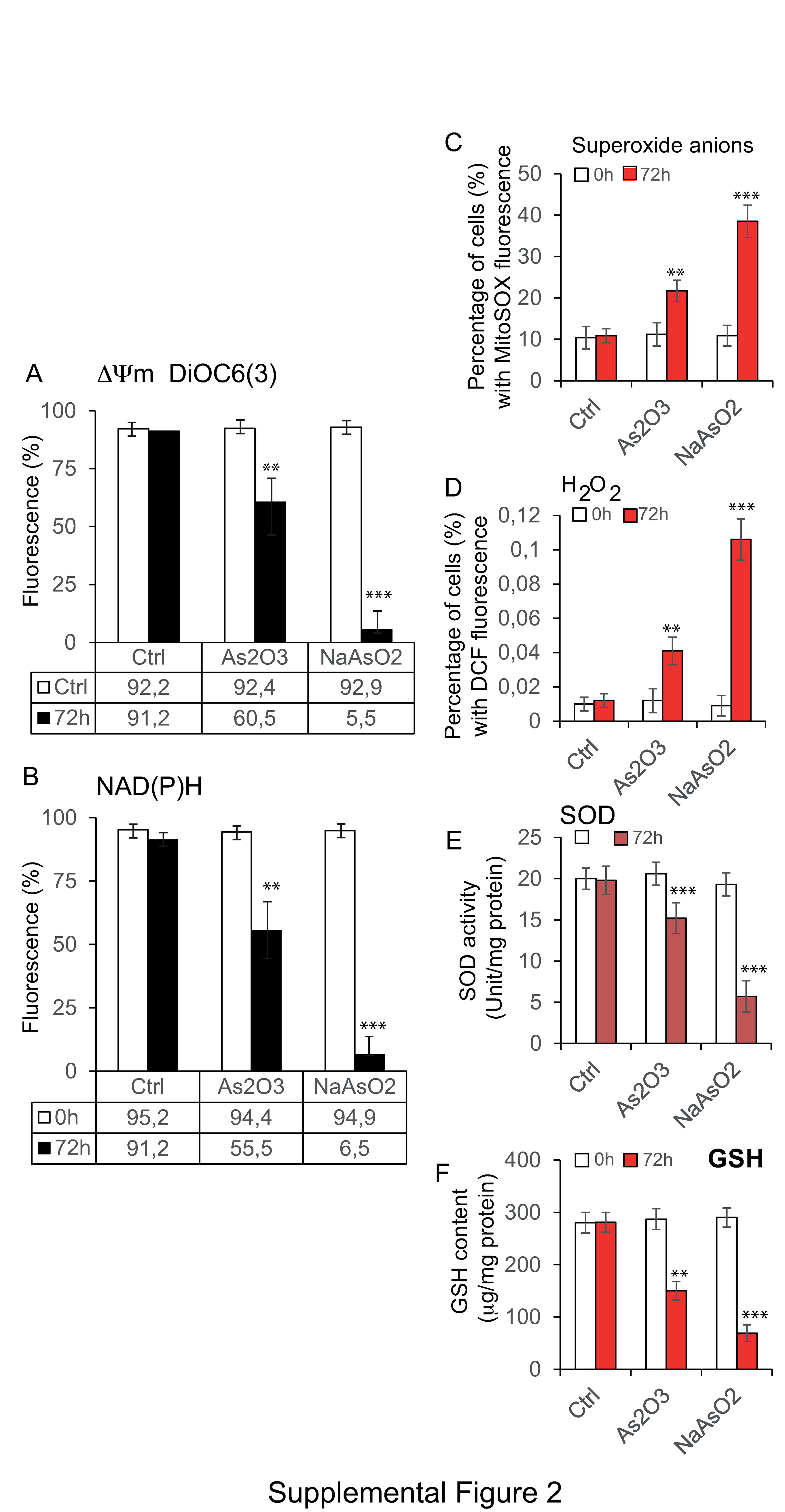

Supplement: S2 Fig — A—Histogram representation of the mitochondrial membrane potential of Ramos-1 cells as detected with DiOC6(3) in flow cytometry (n = 12). B—Histogram representation of the NAD(P)H level detected by flow cytometry (n = 6). C—Superoxide anion production (n = 12). D—Hydroperoxide production (n = 12). E—Superoxide dismutase activity (n = 6). F—Glutathione synthase activity. (TIF) [file pone.0302701.s002.tif]
